# Chinese herbal medicine (“3 medicines and 3 formulations”) for COVID‐19: rapid systematic review and meta‐analysis

**DOI:** 10.1111/jep.13614

**Published:** 2021-09-16

**Authors:** Yangzihan Wang, Trisha Greenhalgh, Jon Wardle, Oxford TCM Rapid Review Team

**Affiliations:** ^1^ Population Health Science Institute Newcastle University Newcastle upon Tyne UK; ^2^ The Nuffield Department of Primary Care Health Sciences University of Oxford Oxford UK; ^3^ National Centre for Naturopathic Medicine Southern Cross University Lismore New South Wales Australia

**Keywords:** COVID‐19, herbal medicine, meta‐analysis, systematic review

## Abstract

**Background:**

To evaluate the evidence behind claims that Chinese Herbal Medicine, specifically “three medicines and three formulations” (3M3F, comprising Jinhua Qinggan, Lianhua Qingwen, Xuebijing, Qingfei Paidu, Huashi Baidu, and Xuanfei Baidu), is an effective treatment for COVID‐19.

**Methods:**

We searched PubMed, MEDLINE and CNKI databases, preprint servers, clinical trial registries and supplementary sources for Chinese‐ or English‐language randomized trials or non‐randomized studies with comparator groups, which tested the constituents of 3M3F in the treatment of COVID‐19 up to September 2020. Primary outcome was change in disease severity. Secondary outcomes included various symptoms. Meta‐analysis (using generic inverse variance random effects model) was performed when there were two or more studies reporting on the same symptom.

**Results:**

Of 607 articles identified, 13 primary studies (6 RCTs and 7 retrospective non‐randomized comparative studies) with 1467 participants met our final inclusion criteria. Studies were small and had significant methodological limitations, most notably potential bias in assessment of outcomes. No study convincingly demonstrated a statistically significant impact on change in disease severity. Eight studies reported sufficiently similar secondary outcomes to be included in a meta‐analysis. Some statistically significant impacts on symptoms, chest CT manifestations, laboratory variables and length of stay were demonstrated, but such findings were sparse and many remain unreplicated.

**Conclusions:**

These findings neither support nor refute the claim that 3M3F alters the severity of COVID‐19 or alleviates symptoms. More rigorous studies are required to properly ascertain the potential role of Chinese Herbal Medicine in COVID‐19.

**Abbreviations**
3M3F: three medicines and three formulations
AE: adverse event
CDC: Centers for Disease Control and Prevention
CHM: Chinese herbal medicine
CI: confidence interval
CNKI: China National Knowledge Infrastructure
COVID: Corona virus disease
CT: computerized tomography
HSBD: Huashi Baidu
JHQG: Jinhua Qinggan granule
LHQW: Lianhua Qingwen
MD: mean difference
PRISMA: Preferred Reporting Items for Systematic Reviews and Meta‐Analyses
QFPD: Qingfei Paidu
RCT: randomized controlled trial
RoB: risk of bias
RR: risk ratio
SMD: standardized mean differences
TCM: Traditional Chinese medicine
WHO: World Health Organization
XBJ: Xuebijing
XFBD: XuanFei Baidu

## 1 INTRODUCTION

China was the first country to be seriously affected by COVID‐19. The first version of the Novel Coronavirus Pneumonia Treatment Plan was published on 16th January 2020,^1^ and the Plan was soon revised into the seventh edition.^2^ From the fourth revision, the Plan included Chinese herbal medicine (CHM) and recommended CHM to be effective to patients with all stages of disease from observation period to critical phase.^3^, ^4^ Six CHM recipes, known as the “3 Medicines and 3 Formulations” (3M3F, [三药三方]), were selected for use.

The “3 Medicines” (*Jinhua Qinggan* granule‐*JHQG*, *Lianhua Qingwen*‐*LHQW* capsule/granule, *Xuebijing*‐*XBJ*) are repurposed existing medicines, used for symptomatic relief of respiratory illnesses including SARS, H1N1 influenza and pneumonia^5^, ^6^, ^7^ . The “3 Formulations” (Lung Cleansing and Detoxifying Decoction, *Qingfei Paidu‐QFPD* decoction, *Huashi Baidu‐HSBD* formula and *XuanFei Baidu‐XFBD* granule) are novel preparations, developed from existing CHM formulas for treatment of COVID‐19.

The 3M3F were claimed to have significant efficacy after observation of population data, and the role of 3M3F in COVID‐19 treatment was officially announced in a Chinese government press conference on 23 March 2020, with promotion as being able to relieve symptoms, and reduce the number of mild of moderate cases progressing to severe cases.^8^ Specific claims included that the compound significantly improves immunological indicators for both mild and severe COVID‐19; that one of the Medicines (*LHQW*) and the three formulations are effective in improving radiologically‐assessed lung infiltrates; that one of the formulations (*XFBD*) improves lymphocyte count by 17% and cure rate by 22%; and that another of the formulations (*HSBD*) reduces the time for viral testing to turn negative and shortens hospital stay by 3 days. One Medicine (*LHQW*) was suggested to have antiviral and anti‐inflammatory effects by inhibiting the SARS‐COV‐2 replication and reducing the pro‐inflammatory cytokines production at the mRNA levels.^9^ These claims were widely reported in the Chinese press and also in Chinese researchers' communication to the WHO that the traditional and complementary medicine unit of the WHO highly appraised the role of 3M3F.^10^

Due to the concise nature of the official statement, the above findings were communicated in concise language with little detail of data supporting claims. However, despite the paucity of available data, 3M3F was readily and significantly implemented into COVID‐19 treatment management. The South China Morning Post reported that over 90% of Chinese COVID‐19 patients had been treated with CHM.^11^ Large quantities of 3M3F were shipped as part of the Chinese government's aid package to other countries such as Italy, Iran and Iraq.^12^, ^13^ Despite unclear evidence of efficacy and some negative press in the West,^14^ they have been distributed by local civic organizations such as the Red Cross and Chinese embassies.^15^ These organizations were taking the lead because regulations limit the official inclusion of 3M3F in many settings outside of China. Nevertheless, the Chinese guidelines have informed national guidelines for traditional medicine use in COVID‐19 in other countries such as Japan and South Korea, which have fully or partially incorporated 3M3F.^16^, ^17^, ^18^ It is critically important to independently review the evidence base behind such claims considering such formally promotion in China and on international stages.

Whilst multiple reviews have reviewed the role of herbal medicine—and CHM specifically—for COVID‐19, none of them look at 3M3F specifically. Independent review is essential to shed light on the debate around the effectiveness of CHM in the COVID‐19 pandemic. As such, our review is the first systematic review to evaluate whether 3M3F improves outcome in COVID‐19 and test the specific efficacy claims outlined above.

## 2 METHODS

This rapid systematic review is reported following the PRISMA checklist. We largely followed Cochrane Interim Rapid Reviews Guidance produced specifically for the COVID‐19 pandemic,^19^ except for tailoring our search to Chinese bibliographic database. Our team included bilingual authors experienced undertaking systematic review tasks in English and Chinese and familiar with both health systems.

### 2.1 Search strategy and selection criteria

In early May 2020, we searched PubMed, MEDLINE and CNKI (China National Knowledge Infrastructure) databases with date restrictions (2019–2020). We used keywords and MeSH terms in domains of COVID (e.g., “COVID‐19”, “Coronavirus”), Chinese and herbal medicine (e.g., “Herbal medicine”, “Traditional Chinese Medicine”), official terms for the 6 Medicines, (e.g., “*Lianhua Qingwen*”) and Chinese, English and botanical terms for individual ingredients associated with the 3 formulations (e.g., “*Ma Huang*”). Using the same or similar keywords, we searched pre‐print servers (MedRxiv and BioRxiv), clinical trial registries (ChiCTR, Clinicaltrials.gov, WHO ICTRP, PROSPERO), as well as Cochrane Task Exchange, Public Health England and a hand‐search of references from selected articles. A detailed search strategy and search term alternatives are available as supporting information; see Supplementary material S1.

The search was repeated in September 2020. Web pages of Chinese Center for Disease Control and Prevention,^20^ National Health Commission of People's Republic of China^21^ and State Administration of Traditional Chinese Medicine^22^ were searched for reference to clinical studies. Studies identified from English (J. W., X. Y. H.) and Chinese databases (Y. W., J. C.) were screened independently.

We included all Chinese‐ and English‐language comparative studies of 3M3F, including randomized controlled trials (RCTs) or non‐randomized studies of interventions. We included any of the 3M3F used separately or together, and alone or in conjunction with other medicines. To be included, a study of any of the three formulations had to report reasonable details of the formulation which were consistent with guidelines from the State Administration of Traditional Chinese Medicine. We included any study on confirmed COVID‐19 patients, including those suspected initially and diagnosed retrospectively. We placed no limitation on age, disease severity or ethnicity (in practice, most participants would have been Chinese).

### 2.2 Quality appraisal of studies

We used the version 2 of the Cochrane Risk of Bias for randomized trials (RoB 2)^23^ and the Newcastle‐Ottawa Scale for non‐randomized studies.^24^ One reviewer extracted data and critically appraised the studies (Y. W., J. W.). A second reviewer double checked (reviewer 4, reviewer 5). Disagreements were resolved by a third reviewer.

### 2.3 Data extraction

Data were extracted by Y. W., reviewer 4, and reviewer 5. for Chinese‐language sources, and by J. W. and reviewer 4 for English‐language sources. We charted the following fields onto a data extraction sheet: geographic location of recruitment, care setting, inclusion criteria including participants' starting disease severity category, age, gender, proportion of immuno‐depression, pre‐existing conditions, and pregnancy status.

### 2.4 Outcome measurements

We predefined a primary outcome domain (“change in disease severity category at the end of treatment”), since this was a major claim at the government press conference. We sought clearly‐defined categories (preferably from guidelines) and used clinically in the study settings.

China standardized definitions of disease severity early in the COVID‐19 outbreak. The Chinese national guideline categorizes disease severity into mild, moderate, severe and critical; the Chinese Center for Disease Control and Prevention has mild, severe and critical categories.^25^ This definition is cited in the US CDC guideline.^26^ In other countries, “triage category” is used in regional or local settings.^27^ Usually, these categorisations take many clinical characteristics into consideration, including vital signs, symptoms, laboratory, and radiographic findings. We did not include “disposition” (e.g., home care or hospital admission) on its own as a marker of disease severity unless the triage criteria were clearly stated. We included categories “dead” and “cured” if the definition of “cured” was clear, and we did not apply time limitations for disease progression or treatment. Only categorisations from studies using the same definition were eligible for meta‐analysis.

We took an emergent approach to secondary outcomes, adjusting our data extraction sheet to reflect outcomes reported in primary studies. Although a disease severity category is already a composite measure, we analysed changes in symptoms separately as secondary outcomes, because of official claims that 3M3F could relieve symptoms. We extracted treatment outcomes of the symptoms reported in COVID‐19 patients.

At the time of this review, there was no international consensus on the outcomes that should be reported when studying COVID‐19, so we extracted non‐symptom outcomes if they were reported in the primary studies; these included laboratory, radiology and healthcare utilization measures. All these outcomes were mentioned in the press conference.^8^

### 2.5 Data analysis

When there were two or more studies reporting on the same outcome measures, we conducted meta‐analysis using RevMan [v5.4]. For continuous variables, because of variability in diagnostic and inclusion criteria, interventions, and length of treatments and follow‐up, a generic inverse variance random effects model was utilized to pool the mean difference (MD) with 95% confidence interval (CI) to incorporate heterogeneity.^28^ When the units of the outcome measures used across studies were not consistent, the effects as standardized mean differences (SMD) were reported. For dichotomous variables, we compared groups using risk ratio (RR) with 95% CI. Heterogeneity was judged moderate when I^2^ > 30%, substantial when I^2^ > 50%, and considerable when I^2^ > 75%.^28^ Potential sources were investigated in a sensitivity analysis if appropriate when interpreting the findings.

## 3 RESULTS

### 3.1 Description of dataset

**Figure 1:**
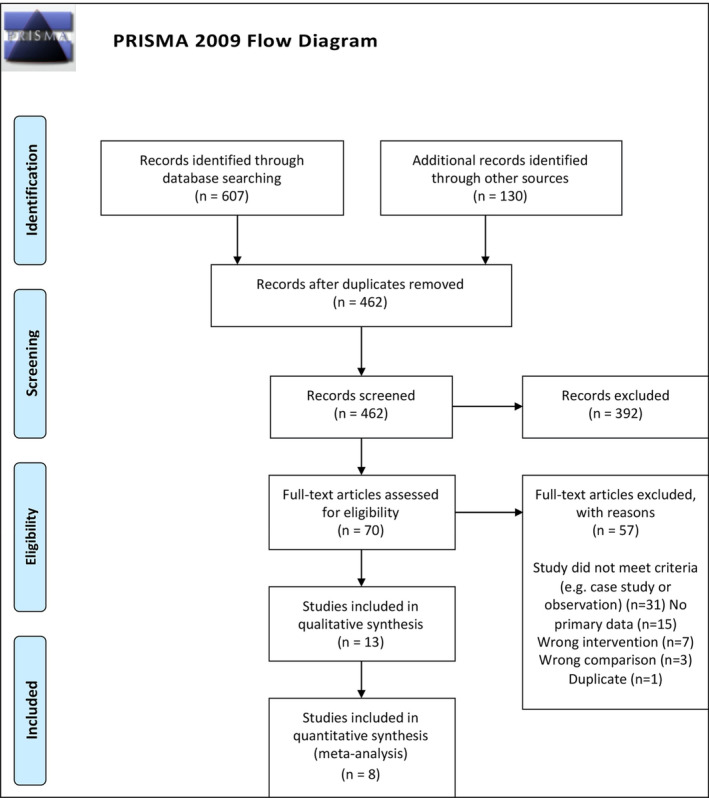
Study selection PRISMA flow chart

The study flowchart is shown in Figure 1. Thirteen studies ‐ Six randomized controlled trials and seven retrospective non‐randomized comparative studies covering a total of 1467 participants ‐ met our final inclusion criteria. All the studies were conducted in China: seven in Wuhan, Hubei; one study^29^ in Qiandongnan, Guizhou; one^30^ in Beijing; one^31^ in Changsha, Hunan; one^32^ in Shiyan and one^33^ in Xiangyang, Hubei; another one^34^ was a large scale of study recruiting patients from 23 hospitals of nine provinces of mainland China. They covered three Medicines (*LHQW*, *JHQG* and *XBJ*) and one formulation (*QFPD* decoction). No relevant study was identified from China CDC, NHC and SATCM's websites.

**Table 1:** Studies reporting treatment of COVID‐19 patients with 3M3F

Component tested	Author/year	Study type	Source	Intervention			Care Setting	Disease severity	Sample size	Mean age	Gender (% female)
				Species, concentration	Quality control reported? (Y/N)	Chemical analysis reported? (Y/N)					
LHQW capsule	Hu 2020	RCT	Yiling (Shijiazhuang & Beijing) Pharmaceutical Co., Ltd., National Medicine Permission No. Z20100040^a^	*Forsythia suspensa* (Thunb.) Vahl 170 g, *Lonicerae Japonicae* Flos. 170 g, honey‐roasted? *Ephedra sinica* 57 g, Fried *Semen Armeniacae Amarum* 57 g, *Gypsum Fibrosum* 170 g, *Isatis tinctoria* L. 170 g, *Rhizoma Dryopteris Crassirhizomae* 170 g, *Houttuynia cordata* Thunb. 170 g, *Pogostemon cablin* (Blanco) Benth. 57 g, *Rheum officinale* Baill. 34 g, *Rhodiola rosea* Linn. 57 g, *Mentholum* 5 g, *Glycyrrhiza uralensis* Fisch 57 g	N^b^	Y^c^	23 hospitals	Mild and moderate	284	TG 50 CG 52	TG 44% CG 50%
LHQW granule	Xiao 2020	RCT (3 arms)			Y^d^	N^e^	Isolation treatment site	Mild and moderate (assume)	193	LHQW TG 55 COMB TG 54 CG 54	LHQW TG 38% COMB TG 51% CG 47%
	Yu 2020	RCT			N^b^	N^e^	Inpatient	Mild and moderate	295	TG 48 CG 47	TG 44% CG 40%
	Cheng 2020	Retrospective cohort			N^b^	N^e^	Inpatient	Moderate	102	TG 56 CG 56	TG 49% CG 47%
	Yao 2020	Retrospective cohort			N^b^	N^e^	Inpatient	Moderate	42	TG 57 CG 62	TG 24% CG 43%
	Lv 2020	Retrospective cohort			N^b^	N^e^	Inpatient	Moderate	101	TG 59 CG 60	TG 56% CG 53%
JHQG decoction	Duan 2020	RCT	Juxiechang (Beijing) Pharmaceutical Co. Ltd., National Medicine Permission No. Z20160001	*Lonicera japonica* Thunb., *Gypsum Fibrosum*, *E. sinica* concocted with honey, Fried *Semen Armeniacae Amarum*, *Astragalus membranaceus*, *Forsythia suspensa* (Thunb.) Vahl, Zhengjiang *Fritillaria thunbergii* Miq., *Anemarrhena asphodeloides Bunge*, *Arctium lappa* L., *Artemisia annua* L., *Mentha haplocalyx* Briq., *Glycyrrhiza uralensis* Fisch.	N	N^f^	Home	Mild	123	TG 52 CG 50	TG 52% CG 44%
	Liu 2020	Retrospective cohort			N	N	Inpatient	Moderate and Severe	80	TG 51 CG 52	TG 52% CG 56%
QFPD decoction	Li 2020	Retrospective cohort	N/A	*Ephedra sinica* 9 g, Baked *Glycyrrhiza uralensis* Fisch. 6 g, *Semen Armeniacae Amarum* 9 g, Raw *Gypsum Fibrosum* 15‐30 g, *Cinnamomum cassia* Presl. 9 g, *Alisma plantago‐aquatica* Linn. 9 g, *Polyporus umbellaru (Pers.) Fr*. 9 g, *Atractylodes Macrocephala* Koidz. 9 g, *Poria cocos* (Schw.) Wolf. 15 g, *Bupleurum chinensis* DC. 16 g, *Scutellaria Baicalensis* Georgi. 6 g, *Pinellia ternata* (Thunb.) Breit. concocted with ginger 9 g, *Zingiber officinale* Rosc. 9 g, *Aster tataricus* Linn. f. 9 g, *Tussilago farfara* L. 9 g, *Belamcanda chinensis* (Linn.) Redouté 9 g, *Asarum sieboldii* Miq. 6 g, *Dioscorea opposita* Thunb. 12 g, *Citrus junos* Sieb. ex Tanaka 6 g, *Citrus aurantium* L. 6 g, *Agastache rugosa* (Fisch. et Mey.) O. Ktze. 9 g	N	N	Inpatient	92% mild or moderate 8% severe	60	TG 54 CG 50	TG 50% CG 57%
	Xin 2020	Retrospective cohort			Y^g^	N	Inpatient	Mild and moderate	63	TG 46 CG 51	TG 54% CG 54%
XBJ injection	Liu 2020	RCT	Hongri (Tianjing) Pharmaceutical Co., Ltd., National Medicine Permission No. Z20040033^h^	*Carthamus tinctorius* L., *Paeonia lactiflora* Pall., *Ligusticum chuanxiong* Hort., *Salvia miltiorrhiza* Bunge., *Angelica sinensis* (Oliv.) Diels	N	N	Inpatient	Mild	20	All participants: 67	All participants: 40%
	Wen 2020	RCT			N	N	Inpatient	Severe	60	50 ml TG 49 100 ml TG 47 CG 48	50 ml TG 45% 100 ml TG 40% CG 55%
	Zhang2020	Retrospective cohort			N	N	Inpatient	Moderate	44	TG 49 CH 46	TG 55% CG 45%

*Note*: The study conducted by Yao et al. (2020) did not report the source of the medication, however Yiling Pharmaceutical is the only company produces this medication. Not reported in article, but the information was found in Pharmacopoeia of the People's Republic of China 2020 (First Part). Ultra‐performance liquid chromatography fingerprint identified 9 of the 32 common peaks were compared with chemical standards, they are Neochlorogenic acid, chlorogenic acid, cryptochlorogenic acid, isoforsythoside A, forsythoside A, quercitrin, isochlorogenic acid C, forsythin, and glycyrrhizic acid. Complied with the provisions of part I of the 2015 edition of the Chinese Pharmacopoeia. Not reported in article, but the information was found in Pharmacopoeia of the People's Republic of China 2020 (First Part) that an active ingredient of Lianhua Qingwen Granule is Forsythin (C_27_H_34_O_11_) for at least 0.69 mg in each bag of the medication. The article reported as “Jianhua Qinggan Granule is based on Maxing Shigan Tang and Yinqiao San. The active ingredients of Yinqiao San including Chlorogenic acid (C_16_H_18_O_9_), Forsythin (C_27_H_34_O_11_), Arctigenin (C_21_H_24_O_6_), Buddlenoid (C_30_H_26_O_13_), Acacetin (C16H12O5), Liquiritigenin (C15H12O4) and so forth. are effective for anti‐virus.” The study conducted by Xin et al. (2020) reported batch number for each herb. The study conducted by Liu et al. (2020) and Wen et al. (2020) did not report the source of the medication, however Hongri Pharmaceutical is the only company produces this medication.

Abbreviations: CG, comparator group; RCT, randomized controlled trial; TG, treatment group.

The key characteristics of the included studies are given in Table 1. A table of excluded studies with reasons for exclusion is given in the Supplementary material S2.

Eleven studies reference China's national guideline (fourth to seventh revisions) to select study participants. The diagnosis criteria evolved in these revisions. The fifth revision published in early February allowed a clinical diagnosis for patients from high‐risk areas (Hubei Province) without laboratory confirmation, if chest imaging was typical. This was later cancelled in the sixth revision. The seventh revision published in early March added antibody test as an option of laboratory tests. Two studies^34^, ^35^ followed the fourth guideline to select patients, one of which^35^ only involved suspected cases. These suspected cases would be considered “clinically diagnosed” if the fifth guideline criteria were applied. Eight studies followed fifth or sixth guideline^29^, ^32^, ^33^, ^36^, ^37^, ^38^, ^39^, ^40^ with confirmation of laboratory testing, and one of them^36^ included a special inclusion requirement of being hospitalized for more than 6 days. One study^39^ recruited both suspected and diagnosed cases according to the seventh treatment guideline, and used epidemiological history, clinical symptoms, CT images and etiological evidence as criteria. Two studies^30^, ^31^ did not mention guideline‐based diagnosis. Two studies captured post‐acute COVID data,^30^, ^33^ while none followed long enough to observe potential chronic COVID symptoms. Eight studies^30^, ^35^, ^36^, ^37^, ^40^, ^41^ provided a breakdown of participants' underlying conditions, most commonly hypertension (ranging from 12.2% to 33.3%), coronary heart disease (2.1%–16.2%), stroke (5.9%–15.9%), diabetes (7.8%–25.6%). Two studies^39^, ^41^ reported a small number of patients with COPD (1.1%–4.9%). One study^41^ included a small number of patients with pre‐existing respiratory disease (chronic obstructive pulmonary disease and tuberculosis, about 3%). Other small proportion of underlying condition reported including chronic kidney and liver disease, cirrhosis, bronchial asthma, hyperlipidaemia and diseases were not specified.

In all studies except one arm in Ref. 39, CHM were used in conjunction with usual care (as recommended in the current version of the Chinese national guideline), and compared with usual care alone. “Usual care” in all the studies included three main approaches: nutrition and supportive treatment, symptomatic treatment and antiviral and antibacterial treatment.

### 3.2 Quality appraisal of included studies

**Figure 2:**
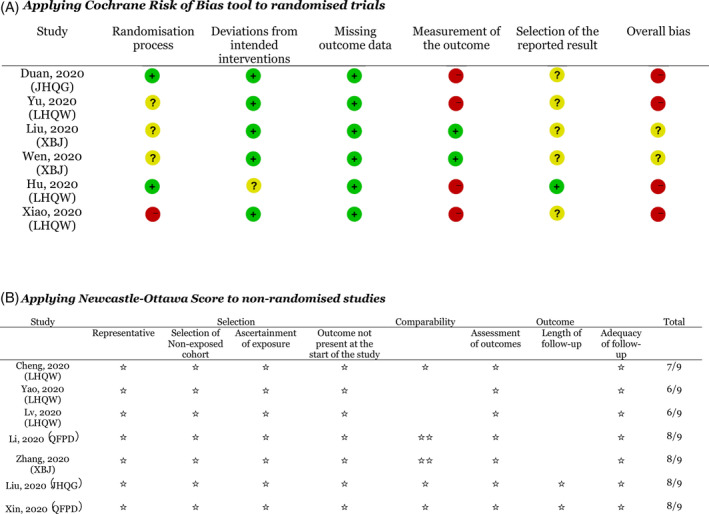
Results of quality appraisal of primary studies

The results of quality appraisal of the included studies are shown in Figure 2.

The quality assessment results of the RCTs are shown in Figure 2(A). There were various forms of concerns for all six trails or they were considered to be at high risk of bias. When evaluating the randomisation process, three trials^34^, ^39^, ^41^ produced random sequences through SPSS or SAS software, whilst random number table was used in another three trials.^29^, ^31^, ^40^ The allocation was concealed in two trials^34^, ^41^ concealed the allocation until the completion of enrolment. Three studies^29^, ^31^, ^40^ did not report allocation concealment. One study^39^ was designed as non‐blind and patients were grouped through a block random method, and this trial was assessed of high risk in the randomisation process. Four trials^34^, ^39^, ^40^, ^41^ were judged to be at high risk of bias in outcome measurement, since assessors' and patients' knowledge of highly promoted interventions could influence assessment on outcomes, such as symptom improvement. The other two RCT^29^, ^31^ were open labelled as well. However, because their main outcomes are derived from laboratory tests, they were judged to be at low risk of bias. Three studies^29^, ^31^, ^40^ did not report whether patients were aware of their allocation. Four studies^29^, ^31^, ^39^, ^41^ reported no trial registration information on the manuscripts. Moreover, incapable of matching the studies with protocols retrieved from Chinese Clinical Trial Registry, we judged them of some concerns with the domain of “selection of reported result”. Only one study^34^ was registered with the Number: Chi CTR‐TRC‐2000029434, but it^30^ did not include intention‐to‐treat analysis which was considered as inappropriate to estimate the effect of assignment to intervention.

Of the non‐randomized studies (all of which were retrospective cohort studies), three studies^35^, ^36^, ^38^ were found to be of fair quality, while the other four studies^30^, ^32^, ^33^, ^37^ were of outstanding quality (Figure 2(B)). There were extensive exclusion criteria for major diseases (including renal disease, cancer and immunodeficiency) in all studies, and comorbid respiratory diseases were excluded in all but one study^41^ . Though the presence of these comorbidities is low for Chinese COVID‐19 patients, the population is likely to be representative of patients with COVID‐19.^42^ The exposed and non‐exposed cohort were from the same community. Two studies^35^, ^38^ failed to be comparable on the basis of study design, and age or disease severity of patients was normally controlled in other studies.^30^, ^32^, ^33^, ^36^, ^37^ All the studies were completed, but only two were considered to be of enough follow‐up length: one study^30^ lasted for 25 days, and clearly‐ recorded data of nucleic acid test and pneumonia recovery situation were collected till the 15th day of hospitalization. Another one^33^ lasted for 22 days. The others five studies^32^, ^35^, ^36^, ^37^, ^38^ were finished within 7–10 days. Overall, medical records were performed in all studies ascertain exposure and did not stipulate the outcome of interest was not stipulated at the beginning of the studies, suggesting a potentially significant source of bias.

### 3.3 Effects of interventions on outcome measures

The included trials featured four comparison groups: *LHQW* (plus usual care) versus usual care (six studies); *XBJ* plus usual care versus usual care (three studies); *JHQG* plus usual care versus usual care (two studies), and *QFPD* plus usual care versus usual care (two studies) (Table 1).

#### 3.3.1 Primary outcome

Our primary outcome measure (change in disease severity category according to clinical guidelines) was adequately reported in only one (non‐randomized) study. One study^36^ reported that there was a significantly lower proportion of patients becoming severe in the treatment group compared to the comparator group, as judged by a *p* value less than 0.05 (see Table 2 for numbers). However, it was based on a small sample size with very few events in some of the cells on the 2 × 2 table. Our own calculation of the data using Fisher exact test, which we believe to be appropriate given the distribution of the data, showed a failure to meet statistical significance (*p =* 0.091) (see Supplementary material S3).

One randomized controlled trial^40^ reported changes in disease severity but we choose not to include these findings because the definition of category used as treatment outcome was not clear. There was also inconsistency in the numbers presented in this study (see Supplementary material S4). Moreover, the study included both mild and moderate patients, but only presented data on progression to severe or dead, missing progression from mild to moderate and progression to critical. We wrote to the corresponding author for clarification, but received no response.

One retrospective analysis^37^ of *QFPD* decoction showed no significant difference in the numbers of patients being cured (as defined by the Chinese national guideline).

**Table 2:** Impact on symptoms: findings from meta‐analysis (green represents that studies suggest a positive benefit; red represents that studies do not suggest a positive benefit)

Covid‐19 symptoms reported	Time reduction (days) (mean difference, 95% CI)	Proportion of patients with symptom resolved ‐ overall 3M3F (risk ratio, 95% CI)	Proportion of patients with symptom resolved ‐ LHQW (risk ratio, 95% CI)	Proportion of patients with symptom resolved ‐ JHQG (risk ratio, 95% CI)
Fever	–0.98 days [−1.78, −0.17]	1.38 [1.19. 1.61]	1.35 [1.14, 1.60]	1.51 [1.07, 2.14]
Cough	—	1.74 [1.31, 2.30]	1.90 [1.24, 2.90]	1.54 [0.97, 2.45]
Fatigue/tiredness	—	1.48 [1.18, 1.86]	1.51 [1.13, 2.00]	1.44 [0.98, 2.11]
Phlegm	—	1.97 [1.08, 3.61]	2.46 [0.81, 7.51]	1.85 [1.01, 3.38]
Short of breath	—	3.93 [1.89, 8.17]	3.93 [1.89, 8.17]	—
Chest tightness	—	2.00 [0.81, 4.96]	2.00 [0.81, 4.96]	—
Diarrhoea	—	1.09 [0.65, 1.82]	1.04 [0.42, 2.58]	1.11 [0.60, 2.07]
Nausea/vomiting	—	1.25 [0.82, 1.90]	1.34 [0.59, 3.06]	1.17 [0.69, 1.99]
Loss in appetite	—	0.63 [0.14, 2.84]	.04 [0.42, 2.58]	0.06 [0.00, 1.03]
Sore throat	—	1.35 [0.68, 2.70]	1.53 [0.38, 6.23]	1.30 [0.58, 2.87]
Headache	—	1.21 [0.83, 1.77]	1.29 [0.67, 2.46]	1.17 [0.73, 1.87]
Muscle pain	—	1.83 [1.02, 3.27]	1.83 [1.02, 3.27]	—
Block/running nose	—	1.00 [0.64, 1.57]	0.90 [0.53, 1.53]	1.31 [0.57, 3.05]

#### 3.3.2 Secondary outcomes

##### Improvement in symptoms

Primary studies measured symptom resolution differently. Fever resolution, for example, was measured in three ways: time taken for fever to resolve, whether fever was resolved after at the end of treatment, and change in symptom score. Assigning a score to a symptom is a common practice in CHM studies, although it has been criticized for systematic errors, non‐standardized use in each study and statistical inappropriateness.^43^ As a result, we will not report on the Traditional Chinese Medicine (TCM) scoring of symptoms, but have included additional information in Supplementary material S5.

**Figure 3:**
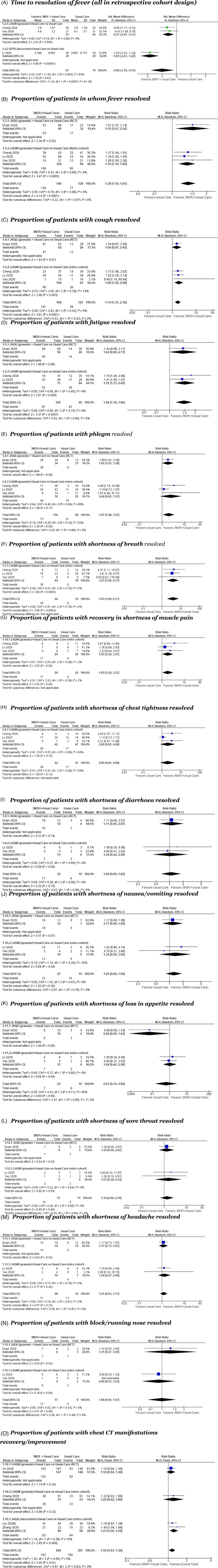
Forest plots of intervention studies where meta‐analysis was possible (findings were interpreted separately)

Figure 3(A‐O) show the results of meta‐analysis of studies which tested the effectiveness of 3M3F on 13 reported COVID‐19 symptoms. Limited findings suggested that 3M3F may reduce time of fever recovery by SMD −0.98 days, 95% CI –1.78 to −0.17; participants = 163; studies = 3; I^2^ = 83%. There were larger proportion of COVID‐19 patients benefited from 3M3F in recovery of fever, cough, fatigue/tiredness, phlegm, short of breath and muscle pain, but not in the other seven symptoms reported (Table 2).

One RCT comparing *LHQW* granule as an add on to antiviral and antimicrobial treatment in line with seventh edition of national guidelines failed to show a reduction in the proportion of patients with improved fever RR 1.00 [0.91, 1.10], cough RR 0.86 [0.69, 1.06], fatigue RR 1.05 [0.84, 1.33], diarrhoea RR 1.00 [0.80, 1.25], nausea/vomiting RR 0.98 [0.75, 1.26], or loss in appetite RR 1.00 [0.80, 1.25], comparing LHQW granule to usual care.^39^

Data from three retrospective cohort studies^36^, ^37^, ^38^ showed a statistically significant effect in favour of 3M3F in reducing time to fever resolution by 0.98 days, 95% CI –1.78 to −0.17; participants = 163; I^2^ = 83%) (Figure 3(A)). Three retrospective cohort studies^35^, ^36^, ^38^ and a single RCT^41^ suggested larger proportion of patients with fever resolved by taking *LHQW* (granule) and JHQG together with usual care RR 1.38, 95% CI 1.19–1.61; participants = 318; I^2^ = 0%) (Figure 3(B)).

There was large heterogeneity among studies reporting the proportion of patients with cough resolved and they showed conflict findings. Three retrospective cohort studies^35^, ^36^, ^38^ favoured *LHQW* group RR 1.90, 95% CI 1.24–2.90; participants = 199; I^2^ = 18%, while a RCT failed to prove the favourable effects of *JHQG* plus usual care versus usual care RR 1.54, 95% CI 0.97–2.45^41^ (Figure 3(C)).

Similar positive findings from RCTs or retrospective cohort studies were observed in the proportion of patients with symptom resolution in fatigue/tiredness (RR 1.48, 95% CI 1.18–1.86; participants = 219; studies = 3; I^2^ = 0%, Figure 3(D)), phlegm (RR 1.97, 95% CI 1.08–3.61; participants = 176; studies = 4; I^2^ = 52%, Figure 3(E)), shortness of breath (RR 3.93, 95% CI 1.89–8.17; participants = 83; studies = 3; I^2^ = 0%, Figure 3(F)), and muscle pain (RR 1.83, 95% CI 1.02–3.27; participants = 49; studies = 3; I^2^ = 2%, Figure 3(G)). On the contrary, studies with small samples failed to show a favourable effect over 3M3F in the resolution of chest tightness (RR 2.00, 95% CI 0.81–4.96; participants = 89; studies = 3; I^2^ = 64%), diarrhoea (RR 1.09, 95% CI 0.65–1.82; participants = 35; studies = 3; I^2^ = 0%), nausea/vomiting (RR 1.25, 95% CI 0.82–1.90; participants = 43; studies = 3; I^2^ = 0%), loss in appetite (RR 0.63, 95% CI 0.14–2.84; participants = 33; studies = 3; I^2^ = 55%), sore throat (RR 1.35, 95% CI 0.68–2.70; participants = 26; studies = 3; I^2^ = 0%), headache (RR 1.21, 95% CI 0.83–1.77; participants = 47; studies = 3; I^2^ = 0%), or block/running nose (RR 1.00, 95% CI 0.64–1.57; participants = 23; studies = 3; I^2^ = 0%).

Table 3 shows the impact on symptom resolution in studies which were not amenable to meta‐analysis. Statistically significant differences were shown for *LHQW* capsule (time to resolution of fever, cough, and fatigue), *LHQW* granule (time to resolution of cough, shortness of breath, symptom scores for fever, dry and sore throat), and *QFPD* decoction (time to resolution of cough).

**Table 3:** Impact on symptoms: findings from analyses not amenable to meta‐analysis

3M3F	*LHQW*				*JHQG*	*QFPD*
Study ID (sample size)	Hu 2020 (n = 284)	Cheng 2020 (n = 102)	Yu 2020 (n = 295)	Lv 2020 (n = 101)	Duan 2020 (n = 123)	Li 2020 (n = 60)
Proportion of patients becoming severe	TG vs. CG 2.1% vs. 4.2%, mean difference: −2.1%, 95%CI: −7.0%–2.4%, *p =* 0.498	TG: 4/51 (7.8%) CG: 11/51 (21.6%) significance discussed in text	Inconsistent definitions and numbers reported		TG: 9/82(11.0%) CG: 10/41(24.4%) *p* > 0.05	TG: 6/30 (20.0%) CG: 12/30 (40.0%), *p* > 0.05
Proportion of patients becoming cured	TG: 91.5%, CG: 82.4%, mean difference: 9.2%, 95%CI 1.3%–17.1%					TG: 27/30 (90.0%) CG: 25/30 (83.3%), *p* > 0.05
Time to resolution of fever (days)	TG vs. CG: 2 vs. 3 days, HR: 1.39, 95%CI: 1.00‐1.94, *p =* 0.017			TG: median 6 d CG: median 7 d *p* = 0.171		
Time to resolution of cough	TG vs. CG: 7 vs. 10 days, HR: 1.71, 95%CI: 1.30‐2.23	TG: 3.9 ± 2.0 CG: 5.2 ± 1.8 *p <* 0.05				TG: 4.9 ± 0.7 days CG: 6.6 ± 0.4 days *p <* 0.05
Time to resolution of fatigue/tiredness (days)	Median (IQR)? TG: 3.0 (3.0‐5.0), CG: 6.0 (4.0‐8.0) HR95%CI 1.8(1.3‐2.5)	TG: 3.5 ± 1.5d, (n = 51); CG: 4.8 ± 1.53 (n = 51) *p =* 0.028 −1.30 [−1.89, −0.71]				

*Note*: All studies' comparator group was usual care; treatment group was usual care plus the component of Chinese herbal medicine. See also Figure 3 for results of meta‐analysis. Apart from one study (Hu et al., 2020) evaluated *LHQW* capsule, all the rest investigated the granule preparation of *LHQW*.

Abbreviations: HR, hazard ratio; IQR, interquartile range.

##### Recovery or improvement of chest CT manifestations

Significant changes were shown in two retrospective cohort studies in time to reduction in lung lesion on CT scan, in *QFPD* (decoction) ‐ 4.80 days, 95% CI –5.82, −3.77, and *JHQG* (decoction) ‐ 0.53 days, 95% CI –0.98, −0.08 at day 15, as adds on to usual care. In addition, there was a larger proportion of patients experiencing recovery/improvement of chest CT manifestations (RR 1.16, 95% CI 1.03–1.30; participants = 521; 3 retrospective cohort studies; I^2^ = 0%, Figure 3(O)).

##### Other secondary outcome measure

Inconclusive findings on blood test results, length of hospital stay, viral conversion, and medication used are reported narratively (Table 4). One non‐randomized study found statistically significant differences in favour of *LHQW* in four laboratory tests (white cell count, lymphocyte count, C‐reactive protein and procalcitonin). The clinical significance of these results is not clear and the authors do not discuss them. Inconclusive findings were observed in reduction in length of stay: one small, non‐randomized study^37^ showed a statistically significant reduction in length of stay in those received *QFPD* decoction, while one^33^ failed to show the same.

**Table 4:** Impact on other secondary outcome measures: findings from analyses not amenable to meta‐analysis

3M3F	*LHQW*			*QFPD*	
Study ID (sample size)	Hu 2020 (n = 284)	Xiao 2020 (n = 188)	Yu 2020 (n = 295)	Li 2020 (n = 60)	Xin 2020 (n = 63)
Proportion of patients whose chest CT improved after 7 days	TG vs. CG: 83.8% vs. 64.1%, mean difference: 19.7%, 95%CI: 9.6%‐29.4%				TG: 1 (1–2), CG: 1 (1‐2) (*p* = 0.482)
White cell count (10̂9/L): pre‐treatment to post‐treatment			TG: 5.1 ± 0.4 to 5.9 ± 0.4, CG: 5.2 ± 0.4 to 5.5 ± 0.4, *p* < 0.05		
Lymphocytes (10̂9/L): pre‐treatment to post‐treatment			TG: 1.5 ± 0.1 to 1.7 ± 0.2, CG: 1.5 ± 0.1 to 1.6 ± 0.2, *p <* 0.05		
C‐reactive protein (mg/L): pre‐treatment to post‐treatment			TG: 26 ± 6 to 22 ± 4 CG: 27 ± 6 to 24 ± 4 *p <* 0.05		
Procalcitonin (ng/L): pre‐treatment to post‐treatment			TG: 0.089 ± 0.025 to 0.058 ± 0.008, CG: 0.094 ± 0.022 to 0.094 ± 0.022 *p <* 0.05		
Length of stay				TG: 13.6 ± 0.4 days, CG: 16.4 ± 0.3 days, *p <* 0.05	TG: 19.0 (15.3‐22.0) days, CG: 17.0 (15.0‐19.3) days, *p* = 0.165
Conversion rate of SAR‐CoV‐2 viral assay	TG vs. CG: 76.8% vs. 71.1%, mean difference: 5.6%, 95%CI: −4.6%‐ 15.7%, *p =* 0.279				
Viral assay conversion time (median)	TG vs. CG: 11.0 vs .12.0 days, HR: 1.21, 95%CI: 0.92‐1.59				
Medications used		Antiviral medication use: TG: 58 (100%); TG COMB: 61 (100%); CG: 63 (100%). Antibiotic use: TG: 30 (51.7%); TG COMB: 25 (41%); CG: 62 (98.4%)			Antibiotic use: *p* = 0.269; Corticosteroid use TG: 7 (18.9%), CG: 5 (19.2%) (*p* = .390); Antiviral drugs Interferon: TG: 34 (91.9%), CG: 26 (100%%), *p* = .140; Arbidol: TG: 24 (64.9%), CG: 16 (61.5%), *p* = 0.997; Lopanivir TG: 29 (78.4%), CG: 25 (96.2%), *p* = 0.049)

*Note*: Apart from one study (Hu et al., 2020) evaluated *LHQW* capsule, all the rest investigated the granule preparation of *LHQW*.

Abbreviation: HR, hazard ratio.

##### Adverse events

No study reported any serious adverse events (AE). Four studies did not discuss AE in their results.^33^, ^36^, ^38^, ^39^ Among those that discussed AEs, three suggested no AE was observed either in the 3M3F or the comparator groups^29^, ^31^, ^35^ and one reported no serious side effects.^40^ One RCT^34^ reported 45.8% (65/142) cases of AEs including abnormal liver function, renal dysfunction, headache, nausea, vomiting, diarrhoea and loss of appetite in the add‐on *LHQW* capsule, while the control group reported 54.2% (77/142) cases with adverse events, including abnormal liver function, renal dysfunction, headache, nausea, vomiting, diarrhoea and loss of appetite. However, such comparison of this study^34^ was found with no statistical significance at 0.84, 95% CI 0.67–1.07. The RCT of^41^ using *JHQG* reported diarrhoea in 27 out of 82 (33%) participants in treatment group versus 0 in control group, and this result has statistically significant difference.

## 4 DISCUSSION

### 4.1 Summary of key findings

Despite strong official endorsement of 3M3F to be effective for COVID‐19, the evidence base for this intervention rests on 13 studies covering a total of 1467 participants. While the limited studies suggest that 3M3F, when used on top of usual care, may offer some relief for some symptoms and changes in lung lesion on CT scan experienced by mostly mild or moderate COVID‐19 patients, the results do not support the high‐level claims that 3M3F could prevent disease from progressing to a more severe type. There were methodological concerns in all studies, with especially high risk of bias in outcomes assessment in the four RCTs. Missing and wrong protocol registration information intensifies our concern over the integrity of these studies.

Of the six remedies making up 3M3F, four had been tested in any experimental study that met our inclusion criteria. Our primary outcome measure (reduction in severity of disease) did not achieve convincing statistical significance in any of the primary studies. In relation to the secondary outcomes, the positive effects of *LHQW*, *JHQG*, and *QFPD* on various symptoms could be explained by bias in assessment of outcome (and in particular, the widespread use of the “symptom score” in TCM), and would need to be replicated before being viewed as definitive. Similarly, the positive impacts of different 3M3F remedies on radiological outcome (two studies), laboratory tests of biomarkers (one study) and length of stay (two studies) need to be replicated before being viewed as definitive.

With the exception of diarrhoea with *JHQG*, the 13 studies did not report any adverse events linked to 3M3F use. Adverse events have, however, been reported in the past when *LHQW* was used for influenza.^44^, ^45^ Previous studies have also reported some digestive system side effects from using *JHQG* to treat influenza, though not significantly more than the control group.^6^ Duan and colleagues attributed the high incidence of diarrhoea in their treatment group to the high dose of *JHQG* they used to treat COVID‐19, and also invoked classical TCM theories to suggest that diarrhoea may have a curative role in this condition.

Although we did not limit the publication language or geography, unsurprisingly all included studies were conducted in China, thus the findings may not be generalisable to other countries. During the editorial process of this manuscript, we noticed a phase three trial of *LHQW* in Singapore was registered, but the results were not posted yet.^46^ There is also no placebo‐controlled study, making it impossible to assess the effect of 3M3F when used alone. Most of the articles are of low quality and sample size, potentially limiting their use in informing practice. We also observe some concerning practices in these studies, for example, the number of trials registered in Clinical Trial Registry is small, and in one case we cannot even find the registered protocol using the protocol number given by the authors. Informed consent was collected only verbally in some studies. However, it should be recognized that these studies were often performed quickly and opportunistically in the early acute phase of a sudden pandemic without proper planning, and some limitations in study design and execution are understandable. Moreover, these issues are not unique to studies of 3M3F. There was a lack of core outcome set for clinic trails of both Western medicine and traditional Chinese medicine during in the early COVID‐19 period, and this led to reporting inconsistencies similar to what we saw in this review.^47^ However, given the limitations of the studies published so far, the results of the studies do not appear to definitively support the claim that 3M3F could prevent the progression of COVID‐19.

### 4.2 Strengths and limitations of this review

To our knowledge, this is the first systematic review and meta‐analysis of a group of CHM specifically promoted for COVID‐19. Whilst some systematic reviews have examined the impact of integrating any CHM with conventional treatment,^48^, ^49^, ^50^ our review has expanded these findings by concentrating on more specific aspects to avoid overgeneralisation. Firstly, comparing with,^48^ we had examined the impact of 3M3F which was explicitly promoted for use in COVID‐19, and till January 2021, our review has identified all the published clinical studies using 3M3F as interventions. Secondly, comparing with,^49^ we included both RCTs and non‐RCTs to provide more comprehensive information to examine the work of 3M3F, because large‐scale of RCTs are insufficient in this field of research and data from other types of studies also works as evidence. Thirdly, comparing with,^49^ we had provided more accurate and detailed information in quality appraisals of included studies and, comparing with,^50^ independent analysis of outcomes of each intervention. We followed Cochrane interim guidance for rapid reviews during this pandemic,^19^ and undertook independent statistical analysis of key findings from primary studies.

One limitation is the small number of primary studies identified. The relative success of China in managing the initial and second waves of COVID‐19 may have limited the ability to conduct trials after detailed protocols based on early clinical experience had been developed. It is also possible that the Chinese government had access to additional unpublished data before developing its official statement on 3M3F. At least 39 clinical trials for CHM interventions were registered in the Chinese Clinical Trials Registry by January 2021 before this review was initiated, though it is unclear how many of these relate to 3M3F.^47^ If such data exist, we recommend that they are placed in the public domain, for example, by sharing and regularly updating data under their registries, to ensure clinicians, researchers and policy‐makers are appropriately informed. Another limitation is that other traditional medicines used for treatment of COVID‐19 were not included in our review. We prioritized 3M3F as it has been officially sanctioned and promoted by the Chinese government for use in China and other foreign countries.

### 4.3 Suggestions for further research

Larger, multi‐centre randomized placebo‐controlled trials of CHM, and especially 3M3F, are urgently needed, with consistent inclusion criteria and objective outcome measures designed to contribute to meta‐analyses. Better reporting of adverse events is needed to confirm the safety profile of 3M3F. It was beyond the scope of this review to explore the pharmaceutical properties and alleged antiviral mechanisms of the various ingredients; there is much scope for further studies in this area, perhaps with a view to developing new chemical entities for mainstream medicine. Many of these studies were performed before much as known about the disease, or which outcomes were most appropriate for inclusion. Only one study attempted to measure or report viral load of COVID‐19 patients or whether this was reduced with the intervention; such variables should be included in further research. Additionally, as our examination focused primarily on the use of CHM in acute COVID‐19 treatment, future research examination of CHM for longer‐term symptomatic relief may be warranted given that many outcomes measured in the studies are also often reported as significant in post‐acute COVID‐19.^51^

## 5 CONCLUSIONS

The findings from this rapid systematic review neither support nor refute the official claim that CHM (specifically 3M3F) alters the severity of COVID‐19 or provides alleviation of symptoms. While the limited studies appear to suggest that 3M3F, when used on top of usual care, may offer some relief for some symptoms experienced by mostly mild or moderate COVID‐19 patients, the results do not support the high‐level claims that 3M3F could prevent disease from progressing to a more severe type. Studies were few in number, small in size, and had significant methodological limitations (most notably, potential bias in assessment of outcomes), though the positive nature of some individual findings do suggest further examination may be warranted. More rigorous multi‐centre randomized placebo‐controlled trials with decent sample sizes are required to properly ascertain the potential role of CHM in treatment of COVID‐19.

## CONFLICT OF INTEREST

The authors declare no conflicts of interest.

## AUTHOR CONTRIBUTIONS

Conceptualisation: All authors. Investigation: All authors. Methodology: All authors. Acquisition of data: Yangzihan Wang, Jon Wardle, reviewer 4, reviewer 5. Formal analysis: All authors. Writing–original draft: All authors. Writing–review and editing: All authors. All authors have read and agreed to the published version of the manuscript.

## Supporting information


**Appendix** **S1**: Supplementary information.

## Data Availability

This review contains secondary analyses of published data. The datasets used and/or analysed during the current study are available from the corresponding author on reasonable request. The data that supports the findings of this study are available in the supplementary materials.
