# Supplementary material for: Chinese herbal medicine (“3 medicines and 3 formulations”) for COVID‐19: rapid systematic review and meta‐analysis
Source: J Eval Clin Pract. 2021 Sep 16;28(1):13–32. doi: 10.1111/jep.13614 (PMC8657519; doi:10.1111/jep.13614)
Supplement: Supplementary file 1 — Appendix S1: Supplementary information. [file JEP-28-13-s001.zip › JEP_13614_3. RR_COVID_3M3F_Supplement v1.0.docx]

**Supplementary Material**

**Supplementary Material 1: *Search strategy***

***PubMed***

1. *Jinhuaqinggan OR “jinhua qinggan” OR “jin hua qing gan”*
2. *Lianhuaqingwen OR “lianhua qingwen” OR “lian hua qing wen”*
3. *Xuebijing OR “xue bi jing”*
4. *Qingfeipaidu OR “qingfei paidu” OR “qing fei pai du” OR “lung cleansing and detoxifying”*
5. *Huashibaidu OR “huashi baidu” OR “hua shi bai du”*
6. *Xuanfeibaidu OR “xuanfei baidu” OR “xuan fei bai du”*
7. *Herbal Medicine/*
8. *Medicine, Chinese Traditional/*
9. *Medicine, East Asian Traditional/*
10. *Plants, Medicinal/*
11. *Drugs, Chinese Herbal/*
12. *chinese herb*.tw.*
13. *(chinese adj2 medicin*).tw.*
14. *(Jinyinhua OR “jin yin hua” OR Shigao OR “shi gao” OR Mahuang OR “ma huang” OR Kuxingren OR “ku xing ren” OR Huangqin OR “huang qin” OR Lianqiao OR “lian qiao” OR Zhebeimu OR “zhe bei mu” OR Zhimu OR “zhi mu” OR Niubangzi OR “niu bang zi” OR Qinghao OR “qing hao” OR Bohe OR “bo he” OR Gancao OR “gan cao” OR Banlangen OR “ban lan gen” OR Mianmaguanzhong “mian ma guan zhong” OR Yuxingcao OR “yu xing cao” OR Guanghuoxiang “Guang huo xiang” OR Dahuang OR “da huang” OR Hongjingtian OR “hong jing tian” OR Bohenao OR “bo he nao” OR Honghua OR “hong hua” OR Chishao OR “chi shao” OR Chuanxiong OR “chuan xiong” OR Danshen OR “dan shen” OR Danggui OR “dang gui” OR Guizhi OR “gui zhi” OR Zexie OR “ze xie” OR Zhuling OR “zhu ling” OR Baizhu OR “bai zhu” OR Fuling OR “fu ling” OR Chaihu OR “chai hu” OR Huangqin OR ”huang qin” OR Banxia OR “ban xia” OR Shengjiang OR “sheng jiang” OR Ziwan OR “zi wan” OR Donghua OR “dong hua” OR Shegan OR “she gan” OR Xixin OR “xi xin” OR Shanyao OR “shan yao” OR Zhishi “zhi shi” OR Chenpi OR “chen pi” OR Huoxiang OR “huo xiang” OR Houpu OR “hou pu” OR Cangzhu OR “cang zhu” OR Caoguo OR “cao guo” OR Tinglizi OR “ting li zi” OR yiyiren OR cangzhu OR “mao cang zhu” OR qinghaocao OR “qing hao cao” OR huzhang OR “hu zhang” OR mabiancao OR “ma bian cao” OR ganlugen OR “gan lu gen” OR huajuhong OR “hua ju hong” OR shenggancao OR “sheng shi gao”)[Title/Abstract]*
15. *“Lonicera japonica” OR “Gypsum fibrosuum” OR Gypsum OR “Calcium Sulfate” OR Ephedra OR “Armeniacae Amarum” OR “Astragalus membranaceus” OR “Astragalus propinquus” OR “Forsythia suspensa” OR “Fritillaria thunbergii” OR “Anemarrhena asphodeloides” OR “Arctium lappa” OR Arctium OR “Artemisia carvifolia” OR “Mentha haplocalyx” OR “Glycyrrhiza uralensis” OR “Isatis tinctorial” OR Dryopteris OR “Dryopteris Crassirhizomae” OR “Houttuynia cordata” OR Pogostemon OR “Pogostemon cablin” OR Rhodiola OR “Rhodiola rosea” OR Menthol OR “Agastache rugosa” OR “Paeoniae lactiflora” OR Paeoniae anomala” OR “Radix Paeoniae Rubra” OR “Conioselinum anthriscoides” OR “Salvia miltiorrhiza” OR “Angelica sinensis” OR “Cinnamomum cassia” OR “Cinnamomum aromaticum” OR Alisma OR “Alisma plantago-aquatica” OR Polyporus OR “Poria cocos” OR Wolfiporia OR “Bupleurum chinensis” OR Pinellia OR “Pinellia ternate” OR Ginger OR “Zingiber officinale” OR “Aster tataricus” OR Tussilago OR “Tussilago farfara” OR “Belamcanda chinensis” OR “Iris domestica” OR “Asarum sieboldii” OR Dioscorea OR “Dioscorea opposite” OR Citrus OR “Citrus junos” OR “Citrus aurantium” OR Magnolia OR “Magnolia officinalis” OR Atractylodes OR “Atractylodes lancea” OR “Amomum tsaoko” OR “Lanxangia tsao-ko” OR “Lanxangia tsaoko” OR Rheum OR “Rheum officinale” OR Coix OR “Coix lacryma-jobi” OR “Reynoutria japonica” OR “Verbena officinalis” OR “ Rhizoma Phragmatis” OR “Phragmitis australis” OR “Semen Lepidii” OR “Descurania Sophia” OR “Lepidium apetalum” OR “Exocarpium Citri Grandis” OR “Citrus maxima” [Title/Abstract]*
16. *or/1-15*
17. *"COVID-19" [Supplementary Concept] OR "COVID-19 drug treatment" [Supplementary Concept] OR "severe acute respiratory syndrome coronavirus 2" [Supplementary Concept]*
18. *“covid-19” OR “covid19”*
19. *“SARS-CoV-2” OR “2019-nCoV” OR “2019nCoV”*
20. *“severe acute respiratory syndrome” AND (“coronavirus” OR “corona virus”)*
21. *((2019 OR 19 OR wuhan OR china) NEAR3 coronavirus) OR ((2019 OR 19 OR wuhan OR china) NEAR3 “corona virus”)*
22. *or/17-21*
23. *16 and 22*

**Supplementary Material 2: Table of excluded studies**

| **Reference** | **Summary Comment for Exclusion** |
| --- | --- |
| Abdelmageed, M. I., et al. "Design of multi epitope-based peptide vaccine against E protein of human COVID-19: An immunoinformatics approach." bioRxiv: 2020.2002.2004.934232. | Inappropriate (bioinformatics) study type and intervention |
| AbdelMassih AF, Ye J, Kamel A et al. A multicenter consensus: A role of furin in the endothelial tropism in obese patients with COVID-19 infection. Obesity Medicine. 2020100281. | Inappropriate (epidemiological) study type and inappropriate intervention |
| Ahmad SI. 5-Fluorouracil in combination with deoxyribonucleosides and deoxyribose as possible therapeutic options for the Coronavirus, COVID-19 infection. Med Hypotheses. 2020 Sep;142:109754. | Not a primary study |
| Afolabi SA, Folorunso SO, Bunyula ZS et al. Social Listening: A Thematic Analysis of COVID-19 Discussion on Social Media. medRxiv. 2020 | Inappropriate (health services research) study type and no intervention |
| Akalin E, Ekici M, Alan Z et al. Traditional Chinese medicine practices used in COVID-19 (Sars-cov 2/Coronavirus-19) treatment in clinic and their effects on the cardiovascular system. Turk Kardiyol Dern Ars. 2020;48:410-424. | Not a primary study |
| Ala’a B, Akour A, Alfalah L. Is It Just About Physical Health? An Online Cross-Sectional Study Exploring the Psychological Distress Among University Students in Jordan in the Midst of COVID-19 Pandemic. Frontiers in Psychology. 2020;11 | Inappropriate (health services research) study type and no intervention |
| Altamimi H, Alahmad Y, Khazal F et al. The Outcome of COVID-19 Patients with Acute Myocardial Infarction. medRxiv. 2020 | Inappropriate (epidemiological) study type and no intervention |
| Alyami HS, Orabi MAA, Aldhabbah FM et al. Knowledge about COVID-19 and beliefs about and use of herbal products during the COVID-19 pandemic: A cross-sectional study in Saudi Arabia. Saudi Pharmaceutical Journal. 2020;28:1326-1332. | Inappropriate (health services research) study type and no intervention |
| Amber R, Adnan M, Tariq A, Mussarat S. A review on antiviral activity of the Himalayan medicinal plants traditionally used to treat bronchitis and related symptoms. Journal of pharmacy and pharmacology. 2017;69:109-122. | Not a primary study |
| An J, Liao X, Xiao T et al. Clinical characteristics of recovered COVID-19 patients with re-detectable positive RNA test. Annals of translational medicine. 2020;8 | Inappropriate study type and no intervention |
| Ang L, Lee HW, Kim A, Lee JA, Zhang J, Lee MS. Herbal medicine for treatment of children diagnosed with COVID-19: A review of guidelines. Complementary therapies in clinical practice. 2020101174. | Not a primary study |
| Ang L, Lee HW, Kim A, Lee MS. Herbal medicine for the management of COVID-19 during the medical observation period: A review of guidelines. Integrative medicine research. 2020100465. | Not a primary study |
| Assefa KT, Gashu AW, Mulualem TD. The impact of COVID-19 infection on maternal and reproductive health care services in governmental health institutions of Dessie town, North-East Ethiopia, 2020 GC. medRxiv. 2020 | Inappropriate (health services research) study type and no intervention |
| Bagheri SH, Asghari A, Farhadi M et al. Coincidence of COVID-19 epidemic and olfactory dysfunction outbreak in Iran. Medical journal of the Islamic Republic of Iran. 2020;34:62. | Inappropriate (epidemiological) study type and no intervention |
| Banerjee A, Santra D, Maiti S. Energetics based epitope screening in SARS CoV-2 (COVID 19) spike glycoprotein by Immuno-informatic analysis aiming to a suitable vaccine development. BioRxiv. 2020 | Inappropriate (non-clinical) study type and no intervention |
| Belhadi D, Peiffer-Smadja N, Lescure F-X, Yazdanpanah Y, Mentré F, Laouénan C. A brief review of antiviral drugs evaluated in registered clinical trials for COVID-19. MedRxiv. 2020 | Not a primary study |
| Bian H, Zheng Z-H, Wei D et al. Meplazumab treats COVID-19 pneumonia: an open-labelled, concurrent controlled add-on clinical trial. MedRxiv. 2020 | Inappropriate intervention (non-TCM) |
| Cai X-f, Chen J, li Hu J et al. A peptide-based magnetic chemiluminescence enzyme immunoassay for serological diagnosis of coronavirus disease 2019. The Journal of infectious diseases. 2020;222:189-193. | Inappropriate (non-clinical) study type and intervention |
| Cao M, Zhang D, Wang Y et al. Clinical features of patients infected with the 2019 novel coronavirus (COVID-19) in Shanghai, China. MedRxiv. 2020 | Inappropriate (epidemiological) study type and no intervention |
| Cao P, Wu S, Wu T, Deng Y, Zhang Q, Wang… K. The important role of polysaccharides from a traditional Chinese medicine-Lung Cleansing and Detoxifying Decoction against the COVID-19 pandemic. Carbohydrate 2020 | Not a primary study |
| Cao R, Hu H, Li Y, Wang X, Xu M, Liu J, Zhang H, Yan Y, Zhao L, Li W, Zhang T, Xiao D, Guo X, Li Y, Yang J, Hu Z, Wang M, Zhong W. Anti-SARS-CoV-2 Potential of Artemisinins In Vitro. ACS Infect Dis. 2020;6(9):2524-2531. | Inappropriate (non-clinical) study type and intervention |
| Chaudhary A, Singh UN, Paudel P, Thapa N, Khadka… K. Characteristics and outcomes of hospitalized adults with COVID-19 in Nepal: a multicenter, prospective cohort study. medRxiv. 2020 | Inappropriate (epidemiological) study type and no intervention |
| Chen B, Liang H, Yuan X et al. Roles of meteorological conditions in COVID-19 transmission on a worldwide scale. MedRxiv. 2020 | Inappropriate (non-clinical) study type and no intervention |
| Chen CJ, Michaelis M, Hsu HK, Tsai… CC. Toona sinensis Roem tender leaf extract inhibits SARS coronavirus replication. Journal of Ethnopharmacol. 2008 | Inappropriate (non-clinical) study type and intervention |
| Chen G, Su W, Yang J, Luo D, Xia P, Jia W, Li X, Wang C, Lang S, Meng Q, Zhang Y, Ke Y, Fan A, Yang S, Zheng Y, Fan X, Qiao J, Lian F, Wei L, Tong X. Chinese herbal medicine reduces mortality in patients with severe and critical Coronavirus disease 2019: a retrospective cohort study. Front Med. 2020 Sep 14:1–8. doi: 10.1007/s11684-020-0813-6. | Inappropriate (epidemiological) study type and no intervention |
| Chen H, Xie Z, Zhu Y, Chen Q, Xie C. Chinese medicine for COVID-19: A protocol for systematic review and meta-analysis. Medicine (Baltimore). 2020 Jun 19;99(25):e20660. | Not a primary study |
| Chen H, Song YP, Gao K, Zhao LT, Ma L. Efficacy and safety of Jinhua Qinggan granules for coronavirus disease 2019 (COVID-19): A protocol of a systematic review and meta-analysis. Medicine (Baltimore). 2020 Jun 12;99(24):e20612. | Not a primary study |
| Chen J, Wang YK, Gao Y, Hu LS, Yang JW, Wang JR, Sun WJ, Liang ZQ, Cao YM, Cao YB. Protection against COVID-19 injury by qingfei paidu decoction via anti-viral, anti-inflammatory activity and metabolic programming. Biomed Pharmacother. 2020 Sep;129:110281.. | Inappropriate (non-clinical) study type |
| Chen KH, Wang SF, Wang SY, Yang YP, Wang ML, Chiou SH, Chang YL. Pharmacological development of the potential adjuvant therapeutic agents against coronavirus disease 2019. J Chin Med Assoc. 2020 Sep;83(9):817-821. | Not a primary study |
| Chen L, Cheng ZQ, Liu F, Xia Y, Chen YG. [Analysis of 131 cases of COVID-19 treated with Ganlu Xiaodu Decoction]. Zhongguo Zhong Yao Za Zhi. 2020 May;45(10):2232-2238. Chinese. | Inappropriate (non-comparator) study type and inappropriate intervention |
| Chen RR, Li YJ, Chen JJ, Lu CL. A review for natural polysaccharides with anti-pulmonary fibrosis properties, which may benefit to patients infected by 2019-nCoV. Carbohydr Polym. 2020 Nov 1;247:116740. | Not a primary study |
| Chen XM, Cao F, Zhang HM, Chen… HR. Exploration of omics mechanism and drug prediction of coronavirus-induced heart failure based on clinical bioinformatics. Zhonghua Xin Xue Guan Bing Za Zhi 48(0): E013. 2020 | Inappropriate (bioinformatics) study type and intervention |
| Chen X, Zheng F, Qing Y et al. Epidemiological and clinical features of 291 cases with coronavirus disease 2019 in areas adjacent to Hubei, China: a double-center observational study. MedRxiv. 2020 | Inappropriate (epidemiological) study type and no intervention |
| Chen Y, Gong X, Wang L, Guo J. Effects of hypertension, diabetes and coronary heart disease on COVID-19 diseases severity: a systematic review and meta-analysis. MedRxiv. 2020 | Not a primary study |
| Chung VCH, Ho LTF, Wu IXY. Chinese medicine diagnosis and treatment for COVID-2019: Is China ready for implementing a national guideline? Adv Integr Med. 2020 May 3. | Not a primary study |
| Ciavarella C, Motta I, Valente S, Pasquinelli G. Pharmacological (or Synthetic) and Nutritional Agonists of PPAR-γ as Candidates for Cytokine Storm Modulation in COVID-19 Disease. Molecules. 2020 Apr 29;25(9):2076. | Not a primary study |
| Ciotti M, Ciccozzi M, Terrinoni A, Jiang WC, Wang CB, Bernardini S. The COVID-19 pandemic. Crit Rev Clin Lab Sci. 2020 Sep;57(6):365-388. | Not a primary study |
| Citkovitz C, Schnyer RN. Chinese Herbal Medicines During the Covid-19 Pandemic: A Role for Observational Studies. J Altern Complement Med. 2020 Jul;26(7):544-546. | Not a primary study |
| China Association Of Integrative Medicine Emergency Medicine C, Editorial Committee Of Chinese Journal Of Integrated Traditional And Western M. [Expert opinion on diagnosis and treatment of coronavirus disease 2019 with traditional Chinese medicine based on "three syndromes and three methods"]. Zhonghua Wei Zhong Bing Ji Jiu Yi Xue. 2020 Jun;32(6):641-645. Chinese. | Not a primary study |
| Coomes EA, Haghbayan H. Interleukin‐6 in COVID‐19: a systematic review and meta‐analysis. Reviews in medical virology. 2020 | Not a primary study |
| Dang JZ, Zhu GY, Yang YJ, Zheng F. Clinical characteristics of coronavirus disease 2019 in patients aged 80 years and older. J Integr Med. 2020 Sep;18(5):395-400. | Inappropriate (epidemiological) study type and no intervention |
| Davoudi-Monfared, E., et al. “Efficacy and safety of interferon beta-1a in treatment of severe COVID-19: A randomized clinical trial” medRxiv 2020.05.28.20116467 | Inappropriate intervention (non-TCM) |
| Dittmar, M., et al “Drug repurposing screens reveal FDA approved drugs active against SARS-Cov-2” bioRxiv 2020.06.19.161042;2020 | Inappropriate (non-clinical) study type and no intervention |
| Dong, M., et al. "Understand Research Hotspots Surrounding COVID-19 and Other Coronavirus Infections Using Topic Modeling." medRxiv: 2020.2003.2026.20044164. | Not a primary study |
| Duan Y, Zhu HL, Zhou C. Advance of promising targets and agents against COVID-19 in China. Drug Discov Today. 2020 May;25(5):810-812. | Not a primary study |
| Esposito A, Palmisano A, Scotti GM, Morelli… MJ. Why is chest CT important for early diagnosis of COVID-19? Prevalence matters. medRxiv. 2020 | Not a primary study |
| Fajgenbaum, D.C., et al. “Treatments administered to the first 9,152 reported cases of COVID19: a systematic review” medRxiv 2020.05.07.20073981. 2020 | Not a primary study |
| Fan AY, et al. Chinese herbal medicine for COVID-19: current evidence with systematic review and meta-analysis. J Integr Med 18(5). Sep 2020; 385-394 | Not a primary study |
| Fan T, Chen Y, Bai Y, Ma F, Wang H, Yang Y, Chen J, Lin Y. [Analysis of medication characteristics of traditional Chinese medicine in treating coronavirus disease-19 based on data mining]. Zhejiang Da Xue Xue Bao Yi Xue Ban. 2020 May 25;49(1):260-269. Chinese. | Not a primary study |
| Fan TT, Cheng BL, Fang XM, Chen YC, Su F. Application of Chinese Medicine in the Management of Critical Conditions: A Review on Sepsis. Am J Chin Med. 2020;48(6):1315-1330. | Not a primary study. Inappropriate topic |
| Fang B, Zhang W, Wu X, Huang T, Li H, Zheng Y, Che J, Sun S, Jiang C, Zhou S, Feng J. Shenhuang granule in the treatment of severe coronavirus disease 2019 (COVID-19): study protocol for an open-label randomized controlled clinical trial. Trials. 2020 Jun 24;21(1):568. | Not a primary study |
| Faye, C., et al. “A dyssymetry in the figures related to the COVID-19 pandemic in the World: What factors explain the difference between Africa and the rest of the World?” medRxiv 2020.05.17.20104687 | Not a primary study |
| Feng Z, Xie Y, Chun L, Li J. [Study on traditional Chinese medicine common syndrome characteristic of coronavirus disease 2019 based on latent structure combined with system clustering analysis]. Zhonghua Wei Zhong Bing Ji Jiu Yi Xue. 2020 May;32(5):537-543. Chinese. | Inappropriate (non-clinical) study type and no intervention |
| Fiore, C., et al. "Antiviral effects of Glycyrrhiza species." Phytother Res. 2008; 22(2): 141-148. | Not a primary study |
| Fonseca, S., et al. “Association between consumption of fermented vegetables and COVID-19 mortality at a country level in Europe” medRxiv 2020.07.06.20147025 | Inappropriate (epidemiological) study type and no intervention |
| Fonseca, S., et al . “Association between consumption of vegetables and COVID-19 mortality at a country level in Europe” medRxiv 2020.07.17.20155846 | Inappropriate (epidemiological) study type and no intervention |
| Fouogue, J.T., et al. “Poor knowledge of COVID-19 and unfavourable perception of the response to the pandemic by healthcare workers at the Bafoussam Regional Hospital (West Region - Cameroon)” medRxiv 2020.08.20.20178970 | Inappropriate (health services research) study type and no intervention |
| Fu, H., et al. "Analysis on the Clinical Characteristics of 36 Cases of Novel Coronavirus Pneumonia in Kunming." medRxiv: 2020.2002.2028.20029173. | Inappropriate (epidemiological) study type and no intervention |
| Fu, H., et al. "Association between Clinical, Laboratory and CT Characteristics and RT-PCR Results in the Follow-up of COVID-19 patients." medRxiv: 2020.2003.2019.20038315. | Inappropriate (epidemiological) study type and no intervention |
| Fu, S., et al. "Virologic and clinical characteristics for prognosis of severe COVID-19: a retrospective observational study in Wuhan, China." medRxiv: 2020.2004.2003.20051763. | Inappropriate (epidemiological) study type and no intervention |
| Fung, K. P., et al."Immunomodulatory activities of the herbal formula Kwan Du Bu Fei Dang in healthy subjects: a randomised, double-blind, placebo-controlled study." Hong Kong Med J 17 Suppl. 2011; 2: 41-43. | Inappropriate population (healthy subjects) and intervention |
| Gajbhiye, R., et al. “Pregnancy outcomes, Newborn complications and Maternal-Fetal Transmission of SARS-CoV-2 in women with COVID-19: A systematic review of 441 cases” medRxiv 2020.04.11.20062356 | Not a primary study |
| Gao, J., et al. “Repurposing low–molecular-weight drugs against the main protease of severe acute respiratory syndrome coronavirus 2” bioRxiv 2020.05.05.079848 | Inappropriate (non-clinical) study type and no intervention |
| Gao K, Song YP, Chen H, Zhao LT, Ma L. Therapeutic efficacy of Qingfei Paidu decoction combined with antiviral drugs in the treatment of corona virus disease 2019: A protocol for systematic review and meta analysis. Medicine (Baltimore). 2020 May 29;99(22):e20489. | Not a primary study |
| Gao, L., et al. "Prognostic value of NT-proBNP in patients with severe COVID-19." medRxiv: 2020.2003.2007.20031575. | Inappropriate intervention (non-TCM) |
| Gao LQ, Xu J, Chen SD. In Silico Screening of Potential Chinese Herbal Medicine Against COVID-19 by Targeting SARS-CoV-2 3CLpro and Angiotensin Converting Enzyme II Using Molecular Docking. Chin J Integr Med. 2020 Jul;26(7):527-532. | Inappropriate (non-clinical) study type |
| Gao Y, et al. Accumulated Clinical Experiences from Successful Treatment of 1377 Severe and Critically Ill COVID-19 Cases. Curr Med Sci. 2020 Aug;40(4):597-601. | Inappropriate (epidemiological) study type and no intervention |
| Gong P, Guo, Y., Li, X., Wang, N., Gu J. Exploring active compounds of Jinhua Qinggan Granules for prevention of novel coronavirus pneumonia (COVID-19) based on network pharmacology and molecular docking. Chinese Traditional and Herbal Drugs. 2020. | Inappropriate (non-clinical) study type and no intervention |
| Gong, Y.-N., et al. "Sequence variation among SARS-CoV-2 isolates in Taiwan." bioRxiv: 2020.2003.2029.014290. | Inappropriate (non-clinical) study type and no intervention |
| Gray PE, Belessis Y. The use of Traditional Chinese Medicines to treat SARS-CoV-2 may cause more harm than good. Pharmacol Res. 2020 Jun;156:104776. | Not a primary study |
| Gu H, Wang ZF, Xie YM. [Ethical review of clinical study on intervention with traditional Chinese medicine in new public health emergencies]. Zhongguo Zhong Yao Za Zhi. 2020 May;45(10):2287-2290. Chinese. | Not a primary study |
| Guo FF, Zhang YQ, Tang SH, Tang X, Xu H, Liu ZY, Huo RL, Li D, Yang HJ. [TCMATCOV--a bioinformatics platform to predict efficacy of TCM against COVID-19]. Zhongguo Zhong Yao Za Zhi. 2020 May;45(10):2257-2264. Chinese. | Inappropriate (non-clinical) study type and no intervention |
| Han, N., et al. “Identification of SARS-CoV-2 induced pathways reveal drug repurposing strategies” bioRxiv 2020.08.24.265496 | Inappropriate (non-clinical) study type and no intervention |
| He T, Qu R, Qin C, Wang Z, Zhang Y, Shao X, Lu T. Potential mechanisms of Chinese Herbal Medicine that implicated in the treatment of COVID-19 related renal injury. Saudi Pharm J. 2020 Sep;28(9):1138-1148. | Inappropriate (non-clinical) study type and no intervention |
| Hill, T. “Comprehensive Systematic Review to Identify putative COVID-19 Treatments: Roles for Immunomodulator and Antiviral Treatments” medRxiv 2020.08.13.20174060 | Not a primary study |
| Ho LTF, Chan KKH, Chung VCH, Leung TH. Highlights of traditional Chinese medicine frontline expert advice in the China national guideline for COVID-19. Eur J Integr Med. 2020 Jun;36:101116. | Not a primary study |
| Ho, T. Y., et al."Emodin blocks the SARS coronavirus spike protein and angiotensin-converting enzyme 2 interaction." Antiviral Res. 2007; 74(2): 92-101. | Inappropriate (non-clinical) study type and intervention |
| Hong, X.-w., et al. "Analysis of early renal injury in COVID-19 and diagnostic value of multi-index combined detection." medRxiv: 2020.2003.2007.20032599. | Inappropriate (epidemiological) study type and no intervention |
| Hou, Z., et al. "Assessment of public attention, risk perception, emotional and behavioural responses to the COVID-19 outbreak: social media surveillance in China." medRxiv: 2020.2003.2014.20035956. | Inappropriate (content analysis) study type and no intervention |
| Hu, C. Analysis of COVID-19 Cases and Public Measures in China. *SN Compr. Clin. Med.* 2020; 2: 1306–1312. | Not a primary study |
| Hu, L., et al. "Risk Factors Associated with Clinical Outcomes in 323 COVID-19 Patients in Wuhan, China." medRxiv: 2020.2003.2025.20037721. | Inappropriate (epidemiological) study type and no intervention |
| Hu, Z., et al. "Clinical Characteristics of 24 Asymptomatic Infections with COVID-19 Screened among Close Contacts in Nanjing, China." medRxiv: 2020.2002.2020.20025619. | Inappropriate (epidemiological) study type and no intervention |
| Huang F, Li Y, Leung EL, Liu X, Liu K, Wang Q, Lan Y, Li X, Yu H, Cui L, Luo H, Luo L. A review of therapeutic agents and Chinese herbal medicines against SARS-COV-2 (COVID-19). Pharmacol Res. 2020 Aug;158:104929. | Not a primary study |
| Huang J, Wu L, Ren X, Wu X, Chen Y, Ran G, Huang A, Huang L, Zhong D. Traditional Chinese medicine for corona virus disease 2019: A protocol for systematic review. Medicine (Baltimore). 2020 Aug 28;99(35):e21774. | Not a primary study |
| Huang Q, Deng X, Li Y, Sun X, Chen Q, Xie M, Liu S, Qu H, Liu S, Wang L, He G, Gong Z. Clinical characteristics and drug therapies in patients with the common-type coronavirus disease 2019 in Hunan, China. Int J Clin Pharm. 2020 Jun;42(3):837-845. | Inappropriate (epidemiological) study type and no intervention |
| Huang ST, Lai HC, Lin YC, Huang WT, Hung HH, Ou SC, Lin HJ, Hung MC. Principles and treatment strategies for the use of Chinese herbal medicine in patients at different stages of coronavirus infection. Am J Cancer Res. 2020 Jul 1;10(7):2010-2031. | Not a primary study |
| Huang, Y., et al. "Clinical characteristics of 36 non-survivors with COVID-19 in Wuhan, China." medRxiv: 2020.2002.2027.20029009. | Inappropriate (epidemiological) study type and no intervention |
| Huang YX, Wang WX, Zhang S, Tang YP, Yue SJ. The database-based strategy may overstate the potential effects of traditional Chinese medicine against COVID-19. Pharmacol Res. 2020 Sep;159:105046. | Not a primary study |
| Huang Y, et al. Treatment strategies of hospitalized patients with coronavirus disease-19. Aging (Albany NY). 2020 Jun 17;12(12):11224-11237. | Inappropriate (epidemiological) study type and no intervention |
| Iftekhar, A., et al. “Behavioral preventive measures and the use of medicines and herbal products among the public in response to Covid-19 in Bangladesh: A cross-sectional study.” medRxiv 2020.08.15.20175513 | Inappropriate intervention (non-TCM) |
| Ji MY, Qi QG, Xi LT, Xiaohua, A RH, Wang J, Li QY, A GL, Li MH. [Analysis on formula of Mongolian medicine for prevention of COVID-19]. Zhongguo Zhong Yao Za Zhi. 2020 Jul;45(13):3013-3019. Chinese. | Inappropriate intervention |
| Jia H, Han Z, Zhang K, et al. Acupuncture and related interventions for anxiety in coronavirus disease 2019: A protocol for systematic review and meta-analysis. *Medicine (Baltimore)*. 2020;99(30):e21317. | Not a primary study |
| Jia, X., et al. "Clinical significance of IgM and IgG test for diagnosis of highly suspected COVID-19 infection." medRxiv: 2020.2002.2028.20029025. | Inappropriate intervention (no intervention, diagnostic) |
| Jiang, R., et al. "Glycyrrhizic acid improves cognitive levels of aging mice by regulating T/B cell proliferation." bioRxiv: 2020.2003.2025.008821. | Inappropriate (animal) study type and intervention |
| Jiang R, Wang K, Mao W, Zhu W, Hu W, Huang L. Chinese herbal experience for the 2019 novel coronavirus. Crit Care. 2020 Jul 21;24(1):451. | Not a primary study |
| Jiang S, Cui Q, Ni B, Chen Y, Tan Y, Chen W, Chen YZ. Databases for facilitating mechanistic investigations of traditional Chinese medicines against COVID-19. Pharmacol Res. 2020 Sep;159:104989. | Not a primary study |
| Jin, C., et al (2020) “A pattern categorization of CT findings to predict outcome of COVID-19 pneumonia” medRxiv 2020.05.19.20107409 | Inappropriate (non-clinical) study type and no intervention |
| Jin L, Xu Y, Yuan H. Effects of four types of integrated Chinese and Western medicines for the treatment of COVID-19 in China: a network meta-analysis. Rev Assoc Med Bras (1992). 2020 Jun;66(6):771-777. | Inappropriate (non-clinical) study type and no intervention |
| Jin YH, et al.; Evidence-Based Medicine Chapter of China International Exchange and Promotive Association for Medical and Health Care (CPAM); Chinese Research Hospital Association (CRHA). Chemoprophylaxis, diagnosis, treatments, and discharge management of COVID-19: An evidence-based clinical practice guideline (updated version). Mil Med Res. 2020 Sep 4;7(1):41. | Not a primary study |
| Kageyama, Y., et al. “Jinhua Qinggan granule, a Chinese herbal medicine against COVID-19, induces rapid changes in the plasma levels of IL-6 and IFN-γ” medRxiv 2020.06.08.20124453 | Inappropriate study population (healthy individuals) |
| Kageyama, Y., et al. “Qing Fei Pai Du Tang, a Chinese multi-herbal medicine formulated against COVID-19, elevates the plasma levels of IL-1β, IL-18, TNF-α, and IL-8” medRxiv 2020.07.13.20146175 | Inappropriate study population (healthy individuals) |
| Kapoor, K. M. and A. Kapoor. "Role of Chloroquine and Hydroxychloroquine in the Treatment of COVID-19 Infection- A Systematic Literature Review." medRxiv: 2020.2003.2024.20042366. | Not a primary study |
| Khan, M. M. A., et al. "COVID-19 infection during pregnancy: a systematic review to summarize possible symptoms, treatments, and pregnancy outcomes." medRxiv: 2020.2003.2031.20049304. | Not a primary study |
| Kim, H. Y., et al. "In vitro inhibition of coronavirus replications by the traditionally used medicinal herbal extracts, Cimicifuga rhizoma, Meliae cortex, Coptidis rhizoma, and Phellodendron cortex." J Clin Virol 2008; 41(2): 122-128. | Inappropriate (non-clinical) study type |
| Khan S, Ali A, Shi H, Siddique R, Shabana, Nabi G, Hu J, Wang T, Dong M, Zaman W, Han G. COVID-19: Clinical aspects and therapeutics responses. Saudi Pharm J. 2020 Aug;28(8):1004-1008. | Inappropriate (epidemiological) study type and intervention |
| Kong Q, Wu Y, Gu Y, Lv Q, Qi F, Gong S, Chen X. Analysis of the molecular mechanism of Pudilan (PDL) treatment for COVID-19 by network pharmacology tools. Biomed Pharmacother. 2020 Aug;128:110316. | Inappropriate (non-clinical) study type |
| Kumar, A., et al. “Efficacy and Safety of Guduchi Ghan Vati in the Management of Asymptomatic COVID-19 infection: An Open Label Feasibility Study” medRxiv 2020.09.20.20198515 | appropriate intervention (non-TCM) |
| Lau, K. M., et al. "Immunomodulatory and anti-SARS activities of Houttuynia cordata." J Ethnopharmacol 2008; 118(1): 79-85. | Inappropriate (non-clinical, animal) study type, population (SARS) and intervention |
| Law S, Leung AW, Xu C. Is the traditional Chinese herb "*Artemisia annua*" possible to fight against COVID-19? Integr Med Res. 2020 Sep;9(3):100474.. | Not a primary study. Inappropriate intervention. |
| Lei, l. and G. Jian-ya. "Clinical characteristics of 51 patients discharged from hospital with COVID-19 in Chongqing，China." medRxiv: 2020.2002.2020.20025536. | Inappropriate (epidemiological) study type and no intervention |
| Lelešius, R., et al. "In vitro antiviral activity of fifteen plant extracts against avian infectious bronchitis virus." BMC Vet Res 2019; 15(1): 178. | Inappropriate (non-clinical) study type |
| Lem, F.F., et al. “Molecular mechanism of action of repurposed drugs and traditional Chinese medicine used for the treatment of patients infected with COVID-19: A systematic scoping review” medRxiv 2020.04.10.20060376 | Not a primary study |
| Li, H., et al. "Forsythoside a inhibits the avian infectious bronchitis virus in cell culture." Phytother Res 2011; 25(3): 338-342. | Inappropriate (non-clinical) study type and intervention |
| Li, J., et al. "Meteorological factors correlate with transmission of 2019-nCoV: Proof of incidence of novel coronavirus pneumonia in Hubei Province, China." medRxiv: 2020.2004.2001.20050526. | Inappropriate (epidemiological) study type and no intervention |
| Li, J., et al. "Sex differences in clinical findings among patients with coronavirus disease 2019 (COVID-19) and severe condition." medRxiv: 2020.2002.2027.20027524. | Inappropriate (epidemiological) study type and no intervention |
| Li H, Zhou Y, Zhang M, Wang H, Zhao Q, Liu J. Updated Approaches against SARS-CoV-2. Antimicrob Agents Chemother. 2020 May 21;64(6):e00483-20 | Not a primary study |
| Li H, Yang L, Liu FF, Ma XN, He PL, Tang W, Tong XK, Zuo JP. Overview of therapeutic drug research for COVID-19 in China. Acta Pharmacol Sin. 2020 Sep;41(9):1133-1140. | Not a primary study |
| Li LJ, Chen X, Yang WN, Xu XM, Lu LY, Wang J, Kong YX, Zheng JH. Traditional Chinese medicine for the treatment of pulmonary fibrosis: A protocol for systematic review and meta-analysis of overview. Medicine (Baltimore). 2020 Jul 31;99(31):e21310. | Not a primary study |
| Li, S., et al. "Symptom combinations associated with outcome and therapeutic effects in a cohort of cases with SARS." Am J Chin Med 2006; 34(6): 937-947. | Inappropriate (epidemiological) study type and no intervention |
| Li Q, Zhu F, Xiao Y, Liu T, Liu X, Wu G, Zhang L. A Primary Mediastinal Large B-Cell Lymphoma Patient With COVID-19 Infection After Intensive Immunochemotherapy: A Case Report. Front Oncol. 2020 May 22;10:924. | Inappropriate (case report) study type and no intervention |
| Li R, Hou Y, Huang J, Pan W, Ma Q, Shi Y, et al. Lianhuaqingwen exerts anti-viral and anti-inflammatory activity against novel coronavirus (SARS-CoV-2). Pharmacological Research. 2020(prepublish). | Inappropriate (non-clinical) study type |
| Liang F, Dong L, Zhou L, Shi Y, Tian L. Traditional Chinese medicine for symptoms of upper respiratory tract of COVID-19: A protocol for systematic review and meta-analysis. Medicine (Baltimore). 2020 Jul 24;99(30):e21320. | Not a primary study |
| Li X, Liu W, He W, Xie Y, Li J. [Meta-analysis of single-group rate of the distribution of traditional Chinese medicine syndromes in 2 139 patients with coronavirus disease 2019]. Zhonghua Wei Zhong Bing Ji Jiu Yi Xue. 2020 Jun;32(6):664-670. Chinese. | Inappropriate (epidemiological) study type and no intervention |
| Li X, et al. Effect of combination antiviral therapy on hematological profiles in 151 adults hospitalized with severe coronavirus disease 2019. Pharmacol Res. 2020 Jun 18;160:105036. | Inappropriate treatment (non-TCM) |
| Li Y, Liu X, Guo L, Li J, Zhong D, Zhang Y, Clarke M, Jin R. Traditional Chinese herbal medicine for treating novel coronavirus (COVID-19) pneumonia: protocol for a systematic review and meta-analysis. Syst Rev. 2020 Apr 8;9(1):75. | Not a primary study |
| Li Y, Li J, Zhong D, Zhang Y, Zhang Y, Guo Y, Clarke M, Jin R. Clinical practice guidelines and experts' consensuses of traditional Chinese herbal medicine for novel coronavirus (COVID-19): protocol of a systematic review. Syst Rev. 2020 Aug 3;9(1):170. | Not a primary study |
| Li Y, Bi L, Li Y, Hu X, Wang Q, Liang X, Yu X, Dong L, Xie Q. The effectiveness and safety of traditional Chinese medicine for the treatment of children with COVID-19. Medicine (Baltimore). 2020 Jul 24;99(30):e21247. | Not a primary study |
| Li Y, Xu H, Lang H, Li J, Bi L, Li Y, Dong L, Zhang L, Liang X, Zhu H. The efficacy and safety of Chinese traditional medicine injections on patients with coronavirus disease 2019: A protocol for systematic review and meta analysis. Medicine (Baltimore). 2020 Jul 31;99(31):e21024. | Not a primary study |
| Liang K, Huang X, Chen H, Qiu L, Zhuang Y, Zou C, Bai Y, Huang Y. Tongue diagnosis and treatment in traditional Chinese medicine for severe COVID-19: a case report. Ann Palliat Med. 2020 Jul;9(4):2400-2407. | Inappropriate (case report) study type and no intervention |
| Liang, Y., et al. "Recovering Mandibular Morphology after Disease with Artificial Intelligence." medRxiv: 2020.2002.2024.20027193. | Inappropriate (epidemiological) study type and no intervention |
| Lin, B., et al. "Epidemiological Trends of Coronavirus Disease 2019 in China." medRxiv: 2020.2003.2013.20035642. | Inappropriate (epidemiological) study type and no intervention |
| Lin WL, Hon KL, Leung KKY, Lin ZX. Roles and challenges of traditional Chinese medicine in COVID-19 in Hong Kong. Hong Kong Med J. 2020 Jun;26(3):268-269. | Not a primary study |
| Ling, C. Q. "Traditional Chinese medicine is a resource for drug discovery against 2019 novel coronavirus (SARS-CoV-2)." J Integr Med 2020; 18(2): 87-88. | Not a primary study |
| Liu M, Ya G, Yuan Y, Yang K, Shi S, Tian J, et al. Efficacy and safety of herbal medicine (Lianhuaqingwen) for treating COVID-19: A systematic review and meta-analysis. Integrative Medicine Research. 2020. | Not a primary study |
| Liu, B. and J. Zhou. "SARS-CoV protease inhibitors design using virtual screening method from natural products libraries." J Comput Chem 2005; 26(5): 484-490. | Inappropriate (bioinformatics) study type and population (SARS) |
| Liu, B., et al. "Persistent SARS-CoV-2 presence is companied with defects in adaptive immune system in non-severe COVID-19 patients." medRxiv: 2020.2003.2026.20044768. | Inappropriate (epidemiological) study type and no intervention |
| Liu D, You Y, Chen Y, Tang S. Efficacy of integrative Traditional Chinese and Western medicine for the treatment of patients infected with 2019 novel coronavirus (COVID-19): A protocol for systematic review and meta analysis. Medicine (Baltimore). 2020 Jul 17;99(29):e20781. | Not a primary study |
| Liu, J., et al. "Epidemiological, Clinical Characteristics and Outcome of Medical Staff Infected with COVID-19 in Wuhan, China: A Retrospective Case Series Analysis." medRxiv: 2020.2003.2009.20033118. | Inappropriate (epidemiological) study type and no intervention |
| Liu, L., et al. "A preliminary study on serological assay for severe acute respiratory syndrome coronavirus 2 (SARS-CoV-2) in 238 admitted hospital patients." medRxiv: 2020.2003.2006.20031856. | Inappropriate (epidemiological) study type and no intervention |
| Liu M, Zhu H, Xiong Q, Zeng Z, Xu X, Ye M, Zeng Y, Hu X, Zhu Y. The efficacy and safety of fire needle therapy for COVID-19: Protocol for a systematic review and meta-analysis. Medicine (Baltimore). 2020 Aug 21;99(34):e21873. | Not a primary study |
| Liu M, Gao Y, Yuan Y, Yang K, Shi S, Tian J, Zhang J. Efficacy and safety of herbal medicine (Lianhuaqingwen) for treating COVID-19: A systematic review and meta-analysis. Integr Med Res. 2021 Mar;10(1):100644. | Not a primary study |
| Liu M, Gao Y, Yuan Y, Yang K, Shi S, Zhang J, Tian J. Efficacy and Safety of Integrated Traditional Chinese and Western Medicine for Corona Virus Disease 2019 (COVID-19): a systematic review and meta-analysis. Pharmacol Res. 2020 Aug;158:104896. | Not a primary study |
| Liu, R., et al. "Association of Cardiovascular Manifestations with In-hospital Outcomes in Patients with COVID-19: A Hospital Staff Data." medRxiv: 2020.2002.2029.20029348. | Inappropriate (epidemiological) study type and no intervention |
| Liu T, Luo S, Libby P, Shi GP. Cathepsin L-selective inhibitors: A potentially promising treatment for COVID-19 patients. Pharmacol Ther. 2020;213:107587. | Inappropriate (non-clinical) study type and no intervention |
| Liu, X., et al. "Chinese herbs combined with Western medicine for severe acute respiratory syndrome (SARS)." Cochrane Database Syst Rev 10(10): Cd004882. 2012. Assessed May 2020 | Not a primary study |
| López-Alcalde J, Yan Y, Witt CM, Barth J. Current State of Research About Chinese Herbal Medicines (CHM) for the Treatment of Coronavirus Disease 2019 (COVID-19): A Scoping Review. J Altern Complement Med. 2020 Jul;26(7):557-570.. | Not a primary study |
| Lu, H. "Drug treatment options for the 2019-new coronavirus (2019-nCoV)." Biosci Trends 2020; 14(1): 69-71. | Inappropriate (bioinformatics) study type |
| Lung, J., et al. “The potential SARS-CoV-2 entry inhibitor” bioRxiv 2020.03.26.009803 | Inappropriate (non-clinical) study type and no intervention |
| Luo, H., et al. "Can Chinese Medicine Be Used for Prevention of Corona Virus Disease 2019 (COVID-19)? A Review of Historical Classics, Research Evidence and Current Prevention Programs." Chin J Integr Med 2020; 26(4): 243-250. | Not a primary study |
| Luo, E., Zhang, D., Luo, H. *et al.* Treatment efficacy analysis of traditional Chinese medicine for novel coronavirus pneumonia (COVID-19): an empirical study from Wuhan, Hubei Province, China. *Chin Med* 2020; 15: 34. | Inappropriate (epidemiological) study type and no intervention |
| Luo X, Ni X, Lin J, Zhang Y, Wu L, Huang D, Liu Y, Guo J, Wen W, Cai Y, Chen Y, Lin L. The add-on effect of Chinese herbal medicine on COVID-19: A systematic review and meta-analysis. Phytomedicine. 2020 Jul 11:153282. | Not a primary study |
| Ma Q, Qiu M, Zhou H, et al. The study on the treatment of Xuebijing injection (XBJ) in adults with severe or critical Corona Virus Disease 2019 and the inhibitory effect of XBJ against SARS-CoV-2. *Pharmacol Res*. 2020;160:105073. | Not a comparative study |
| Ma, K.-L., et al. "COVID-19 Myocarditis and Severity Factors： An Adult Cohort Study." medRxiv: 2020.2003.2019.20034124. | Inappropriate (epidemiological) study type and no intervention |
| Ma S, Zhang X, Cen J, Hong G, Hong S, Ju W. [A systematic pharmacological investigation of pharmacologically active ingredients in Toujie Quwen granules for treatment of COVID-19]. Nan Fang Yi Ke Da Xue Xue Bao. 2020 Aug 30;40(8):1072-1080. Chinese. | Inappropriate (non-clinical) study type |
| Malik, A.A. “COVID-19 Risk Perception Among U.S. Adults: Changes from February to May 2020”. medRxiv 2020.08.20.20178822 | Inappropriate (health services research) study type and no intervention |
| McFadden, S. M., et al. "Perceptions of the Adult US Population regarding the Novel Coronavirus Outbreak." medRxiv: 2020.2002.2026.20028308. | Inappropriate (survey) study type and no intervention |
| Mirzaie A, Halaji M, Dehkordi FS, Ranjbar R, Noorbazargan H. A narrative literature review on traditional medicine options for treatment of corona virus disease 2019 (COVID-19). Complement Ther Clin Pract. 2020 Aug;40:101214.. | Not a primary study |
| Misra, S., et al. “Effect of various treatment modalities on the novel coronavirus (nCOV-2019) infection in humans: a systematic review & meta-analysis” medRxiv 2020.05.24.20111799 | Not a primary study |
| Nandan, A., et al. “Exploring alternative medicine options for the prevention or treatment of coronavirus disease 2019 (COVID-19)- A systematic scoping review”. medRxiv 2020.05.14.20101352 | Not a primary study |
| Ni L, Chen L, Huang X, Han C, Xu J, Zhang H, Luan X, Zhao Y, Xu J, Yuan W, Chen H. Combating COVID-19 with integrated traditional Chinese and Western medicine in China. Acta Pharm Sin B. 2020 Jul;10(7):1149-1162.. | Not a primary study |
| Nie, Q. H., et al. "Current status of severe acute respiratory syndrome in China." World J Gastroenterol 2003; 9(8): 1635-1645. | Not a primary study |
| Nie, R., et al. "Clinical features and the maternal and neonatal outcomes of pregnant women with coronavirus disease 2019." medRxiv: 2020.2003.2022.20041061. | Inappropriate (epidemiological) study type and no intervention |
| Ngo, B.T., et al. “A systematic analysis of the time course to develop treatments for COVID-19”. medRxiv 2020.05.27.20115238 | Inappropriate (non-clinical) study type and no intervention |
| Olapegba, P.O., et al. “A Preliminary Assessment of Novel Coronavirus (COVID-19) Knowledge and Perceptions in Nigeria” medRxiv 2020.04.11.20061408 | Inappropriate (health services research) study type and no intervention |
| Otitoloju, A.A., et al. “Preliminary evaluation of COVID-19 disease outcomes, test capacities and management approaches among African countries.” medRxiv 2020.05.16.20103838 | Inappropriate (health services research) study type and no intervention |
| Pan HD, Yao XJ, Wang WY, Lau HY, Liu L. Network pharmacological approach for elucidating the mechanisms of traditional Chinese medicine in treating COVID-19 patients. Pharmacol Res. 2020 Sep;159:105043. | Inappropriate (network pharmacology) study type and no intervention |
| Philip, R.K., et al. “Reduction in preterm births during the COVID-19 lockdown in Ireland: a natural experiment allowing analysis of data from the prior two decades.” medRxiv 2020.06.03.20121442 | Inappropriate (epidemiological) study type and no intervention |
| Pu, H., et al. "Screening and managing of suspected or confirmed novel coronavirus (COVID-19) patients: experiences from a tertiary hospital outside Hubei province." medRxiv: 2020.2003.2020.20038679. | Inappropriate (epidemiological) study type and no intervention |
| Pu, J. Y., et al. "[Anti-virus research of triterpenoids in licorice]." Bing Du Xue Bao 2013; 29(6): 673-679. | Inappropriate (non-clinical) study type and intervention |
| Qi, D., et al. "Epidemiological and clinical features of 2019-nCoV acute respiratory disease cases in Chongqing municipality, China: a retrospective, descriptive, multiple-center study." medRxiv: 2020.2003.2001.20029397. | Inappropriate (epidemiological) study type and no intervention |
| Qi, X., et al. "Machine learning-based CT radiomics model for predicting hospital stay in patients with pneumonia associated with SARS-CoV-2 infection: A multicenter study." medRxiv: 2020.2002.2029.20029603. | Inappropriate (bioinformatics) intervention |
| Qian, M., et al. "Psychological responses, behavioral changes and public perceptions during the early phase of the COVID-19 outbreak in China: a population based cross-sectional survey." medRxiv: 2020.2002.2018.20024448. | Inappropriate (epidemiological) study type and no intervention |
| Qing GC, Zhang H, Bai Y, Luo Y. Traditional Chinese and Western Medicines Jointly Beat COVID-19 Pandemic. Chin J Integr Med. 2020 Jun;26(6):403-404. | Not a primary study |
| Qinhai M, Minshan Q, Hongxia Z, Jie C, Xue Y, Zhenxuan D, et al. The study on the treatment of Xuebijing injection (XBJ) in adults with severe or critical Corona Virus Disease 2019 and the inhibitory effect of XBJ against SARS-CoV-2. Pharmacological research. 2020;160. | Non-clinical research (letter) |
| Qiu, C., et al. "Transmission and clinical characteristics of coronavirus disease 2019 in 104 outside-Wuhan patients, China." medRxiv: 2020.2003.2004.20026005. | Inappropriate (epidemiological) study type and no intervention |
| Qiu, R., et al. "Core Outcome Set for Traditional Chinese and Western Medicine Clinical Trials of COVID-19." medRxiv: 2020.2003.2023.20041533. | Not a primary study |
| Qiu, R., et al. "Outcome reporting from protocols of clinical trials of Coronavirus Disease 2019 (COVID-19): a review." medRxiv: 2020.2003.2004.20031401. | Not a primary study |
| Qiu, Y., et al. "Immunopotentiating effects of four Chinese herbal polysaccharides administered at vaccination in chickens." Poult Sci 2007; 86(12): 2530-2535. | Inappropriate (animal) study type |
| Quan, w., et al. "No SARS-CoV-2 in expressed prostatic secretion of patients with coronavirus disease 2019: a descriptive multicentre study in China." medRxiv: 2020.2003.2026.20044198. | Inappropriate (epidemiological) study type and no intervention |
| Quandt, S.A., et al. “COVID-19 Pandemic among Latinx Farmworker and Non-farmworker Families in North Carolina: Knowledge, Risk Perceptions, and Preventive Behaviors” medRxiv 2020.07.14.20153429 | Inappropriate (health services research) study type and no intervention |
| Qureshi, A., et al. "Evaluation of the awareness level of Healthcare workers toward NCOVID-2019 in Pakistan." medRxiv: 2020.2003.2026.20044636. | Inappropriate (survey) study type and no intervention |
| Ren, Jun-Ling et al. “Traditional Chinese medicine for COVID-19 treatment.” *Pharmacological research* 2020; 155 | Inappropriate (case study) study type and inappropriate intervention |
| Ren X, Shao XX, Li XX, Jia XH, Song T, Zhou WY, Wang P, Li Y, Wang XL, Cui QH, Qiu PJ, Zhao YG, Li XB, Zhang FC, Li ZY, Zhong Y, Wang ZG, Fu XJ. Identifying potential treatments of COVID-19 from Traditional Chinese Medicine (TCM) by using a data-driven approach. J Ethnopharmacol. 2020 Aug 10;258:112932. | Not a primary study |
| Rios, P., et al. "Effectiveness and safety of antiviral or antibody treatments for coronavirus: A rapid review." medRxiv: 2020.2003.2019.20039008. | Not a primary study |
| Runfeng, L., et al. "Lianhuaqingwen exerts anti-viral and anti-inflammatory activity against novel coronavirus (SARS-CoV-2)." Pharmacol Res: 104761. | Inappropriate (non-clinical) study type |
| Saddik, B., et al. “Assessing the influence of parental anxiety on childhood anxiety during the COVID-19 pandemic in the United Arab Emirates” medRxiv 2020.06.11.20128371 | Inappropriate (health services research) study type and intervention |
| Schneiderová, K. and K. Šmejkal. "Phytochemical profile of Paulownia tomentosa (Thunb). Steud." Phytochem Rev 2015; 14(5): 799-833. | Inappropriate (non-clinical) study type and intervention |
| Schwarz, S., et al. "Kaempferol derivatives as antiviral drugs against the 3a channel protein of coronavirus." Planta Med 2014; 80(2-3): 177-182. | Inappropriate (non-clinical) study type and intervention |
| Seng, J.J.B., et al. “Pandemic related Health literacy - A Systematic Review of literature in COVID-19, SARS and MERS pandemics” medRxiv 2020.05.07.20094227 | Not a primary study |
| Shahzad F, Anderson D, Najafzadeh M. The Antiviral, Anti-Inflammatory Effects of Natural Medicinal Herbs and Mushrooms and SARS-CoV-2 Infection. Nutrients. 2020 Aug 25;12(9):2573. | Not a primary study |
| Shankar A, Dubey A, Saini D, Prasad CP. Role of Complementary and Alternative Medicine in Prevention and Treatment of COVID-19: An Overhyped Hope. Chin J Integr Med. 2020 Aug;26(8):565-567. | Not a primary study |
| Shi, Q., et al. "Clinical characteristics of 101 non-surviving hospitalized patients with COVID-19: A single center, retrospective study." medRxiv: 2020.2003.2004.20031039. | Inappropriate (epidemiological) study type and no intervention |
| Shi Y, Wang G, Cai XP, Deng JW, Zheng L, Zhu HH, Zheng M, Yang B, Chen Z. An overview of COVID-19. J Zhejiang Univ Sci B. 2020 May;21(5):343-360. | Not a primary study |
| Song, G., et al. ” Advance of Novel Coronavirus Registration Clinical Trial” medRxiv 2020.03.16.20034934 | Not a primary study |
| Song, P. X., et al. "An epidemiological forecast model and software assessing interventions on COVID-19 epidemic in China." medRxiv: 2020.2002.2029.20029421. | Inappropriate (epidemiological) study type and no intervention |
| Song P, et al. Interpretation of the Traditional Chinese Medicine portion of the diagnosis and treatment protocol for corona virus disease 2019 (Trial Version 7). J Tradit Chin Med. 2020 Jun;40(3):497-508. | Not a primary study |
| Song Y, Zhang M, Yin L, Wang K, Zhou Y, Zhou M, Lu Y. COVID-19 treatment: close to a cure? A rapid review of pharmacotherapies for the novel coronavirus (SARS-CoV-2). Int J Antimicrob Agents. 2020 Aug;56(2):106080. | Not a primary study |
| Su, H., et al. “Discovery of baicalin and baicalein as novel, natural product inhibitors of SARS-CoV-2 3CL protease *in vitro*” bioRxiv 2020.04.13.038687 | Inappropriate (non-clinical) study type and intervention |
| Tahvildari, A., et al. "Clinical features, Diagnosis, and Treatment of COVID-19: A systematic review of case reports and case series." medRxiv: 2020.2003.2028.20046151. | Not a primary study |
| Tai, Z.Y., et al. “Mixed Chinese herbs and Western medicine for novel coronavirus disease 2019 (COVID-19): a mixed method review” medRxiv 2020.05.11.20098111 | Not a primary study |
| Takeda Y, Murata T, Jamsransuren D, Suganuma K, Kazami Y, Batkhuu J, Badral D, Ogawa H. Saxifraga spinulosa-Derived Components Rapidly Inactivate Multiple Viruses Including SARS-CoV-2. Viruses. 2020 Jun 28;12(7):699. | Inappropriate (non-clinical) study type and inappropriate intervention |
| Tang, A., et al. "A retrospective study of the clinical characteristics of COVID-19 infection in 26 children." medRxiv: 2020.2003.2008.20029710. | Inappropriate (epidemiological) study type and no intervention |
| Tang X, Tong L, Guo FF, Tang SH, Yang HJ. [Analysis of potential role of Chinese classic prescriptions in treatment of COVID-19 based on TCMATCOV platform]. Zhongguo Zhong Yao Za Zhi. 2020 Jul;45(13):3028-3034. Chinese. | Inappropriate (non-clinical) study type |
| Tang, Z. H., et al. "[Construction of MHV-A59 damp-heat mouse model and analysis of the relevant indices]." Nan Fang Yi Ke Da Xue Xue Bao 2010; 30(11): 2452-2454. | Inappropriate (non-clinical) study type and no intervention |
| Tao LT, Huang TL, Zheng DW, Zou X. Case of professor Xu ZOU's acupuncture technique for "benefiting kidney and strengthening anti-pathogenic *qi*" in promoting the absorption of COVID-19. World J Acupunct Moxibustion. 2020 Oct;30(3):167-170. | Not a primary study |
| Tao Q, Du J, Li X, Zeng J, Tan B, Xu J, Lin W, Chen XL. Network pharmacology and molecular docking analysis on molecular targets and mechanisms of Huashi Baidu formula in the treatment of COVID-19. Drug Dev Ind Pharm. 2020 Aug;46(8):1345-1353. | Inappropriate (non-clinical) study type |
| Tian, S., et al. “Epidemiological investigation and intergenerational clinical characteristics of 24 COVID-19 patients associated with supermarket cluster” medRxiv 2020.04.11.20058891 | Inappropriate (epidemiological) study type and no intervention |
| Tian, S., et al. "Clinical characteristics and reasons of different duration from onset to release from quarantine for patients with COVID-19 Outside Hubei province, China." medRxiv: 2020.2003.2021.20038778. | Inappropriate (epidemiological) study type and no intervention |
| Tong, T., Wu, Y., Ni, W. *et al.* The potential insights of Traditional Chinese Medicine on treatment of COVID-19. *Chin Med* 2020; 15: 51 | Not a primary study |
| Tsai, Y. C., et al. "Antiviral Action of Tryptanthrin Isolated from Strobilanthes cusia Leaf against Human Coronavirus NL63." Biomolecules 10(3). 2020 | Inappropriate (non-clinical) study type and intervention |
| ul Qamar, M. T., et al. "Designing of a next generation multiepitope based vaccine (MEV) against SARS-COV-2: Immunoinformatics and in silico approaches." bioRxiv: 2020.2002.2028.970343. | Inappropriate (non-clinical, bioinformatics) study type and no intervention |
| Venkatselu, B.P., et al. “Mechanistic rationale of drugs, Primary endpoints, Geographical distribution of clinical trials against Severe acute respiratory syndrome-related coronavirus-2: A Systematic Review” medRxiv 2020.05.24.20112169 | Not a primary study |
| Vivek-Ananth RP, Rana A, Rajan N, Biswal HS, Samal A. In Silico Identification of Potential Natural Product Inhibitors of Human Proteases Key to SARS-CoV-2 Infection. Molecules. 2020 Aug 22;25(17):3822. | Inappropriate (non-clinical) study type and no intervention |
| Wang C, Ming H, Jia W, Su W, Zhan LR, Luo D, Yang JY. [Analysis of medication regularity and pharmacodynamic characteristics of traditional Chinese medicine treatment in 444 severe cases of COVID-19]. Zhongguo Zhong Yao Za Zhi. 2020 Jul;45(13):3007-3012. Chinese. | Inappropriate (epidemiological) study type and no intervention |
| Wang, H., et al. “Progression, recovery and fatality in patients with SARS-CoV-2 related pneumonia in Wuhan, China: a single-centered, retrospective, observational study” medRxiv 2020.05.12.20099739 | Inappropriate (epidemiological) study type and no intervention |
| Wang J, Zhu X, Sun Y, Zhang X, Zhang W. Efficacy and safety of traditional Chinese medicine combined with routine western medicine for the asymptomatic novel coronavirus disease (COVID-19): A Bayesian network meta-analysis protocol. Medicine (Baltimore). 2020 Aug 28;99(35):e21927. | Not a primary study |
| Wang L, Xu X, Ruan J, Lin S, Jiang J, Ye H. Quadruple therapy for asymptomatic COVID-19 infection patients. Expert Rev Anti Infect Ther. 2020 Jul;18(7):617-624. | Inappropriate intervention |
| Wang LX, Xie YM. [Suggestions on design of evidence-based traditional Chinese medicine clinical study for new public health emergencies]. Zhongguo Zhong Yao Za Zhi. 2020 May;45(10):2291-2295. Chinese. | Not a primary study |
| Wang Q, Wang C, Mei J, He L, Li J, Liu S, Chen F. [Understanding and prevention of D-dimer elevation in coronavirus disease 2019 in traditional Chinese medicine]. Zhonghua Wei Zhong Bing Ji Jiu Yi Xue. 2020 May;32(5):622-624. Chinese.. | Inappropriate (non-clinical) study type |
| Wang RQ, Liu JX, Zhang ZD, Wen J, Han P, Wu HH, Jia YJ, Jia CS, Pan LJ. [Feasibility analysis on acupuncture therapy for the treatment of Corona Virus Disease 2019 and the exploration on the application scheme]. Zhen Ci Yan Jiu. 2020 May 25;45(5):345-50. Chinese. | Inappropriate intervention |
| Wang S, Zeng X, Wang Y, Zhao Y, Chen W, Chen YZ. East meets West in COVID-19 therapeutics. Pharmacol Res. 2020 Sep;159:105008. | Not a primary study |
| Wang SX, Wang Y, Lu YB, Li JY, Song YJ, Nyamgerelt M, Wang XX. Diagnosis and treatment of novel coronavirus pneumonia based on the theory of traditional Chinese medicine. J Integr Med. 2020 Jul;18(4):275-283.. | Not a primary study |
| Wang, Y., et al. “Organizing Pneumonia of COVID-19: Time-dependent Evolution and Outcome in CT Findings” medRxiv 2020.05.22.20109934 | Inappropriate study type and no intervention |
| Wang, S., et al. “Luteolin transforms the BMDM polarity to regulate the expression of inflammatory factors” bioRxiv 2020.06.30.181503 | Inappropriate (non-clinical) study type and intervention |
| Wang, X., et al. "Estimating the case fatality ratio of the COVID-19 epidemic in China." medRxiv: 2020.2002.2017.20023630. | Inappropriate (epidemiological) study type and no intervention |
| Wang X, Xie P, Sun G, Deng Z, Zhao M, Bao S, Zhou Y. A systematic review and meta-analysis of the efficacy and safety of western medicine routine treatment combined with Chinese herbal medicine in the treatment of COVID-19. Medicine (Baltimore). 2020 Aug 7;99(32):e21616. | Not a primary study |
| Wang Y, Li X, Zhang JH, Xue R, Qian JY, Zhang XH, Zhang H, Liu QQ, Fan XH, Cheng YY, Zhang BL. [Mechanism of Xuanfei Baidu Tang in treatment of COVID-19 based on network pharmacology]. Zhongguo Zhong Yao Za Zhi. 2020 May;45(10):2249-2256. Chinese. | Inappropriate (network pharmacology) study type |
| Wang ZC, et al. Intra-Rater and Inter-Rater Reliability of Tongue Coating Diagnosis in Traditional Chinese Medicine Using Smartphones: Quasi-Delphi Study. JMIR Mhealth Uhealth. 2020 Jul 9;8(7):e16018. doi: 10.2196/16018. PMID: 32459647; PMCID: PMC7380897. | Inappropriate topic |
| Wang ZF, Wang YP, Zhang HM, Fan YP, Lü C, Wang YY. [Thinking on Clinical rational use of TCM injection in the treatment of novel coronavirus pneumonia (COVID-19)]. Zhonghua Yi Xue Za Zhi. 2020 Mar 3;100(0):E016. Chinese. | Not a primary study |
| Wassenaar TM, Zou Y. 2019_nCoV/SARS-CoV-2: rapid classification of betacoronaviruses and identification of Traditional Chinese Medicine as potential origin of zoonotic coronaviruses. Lett Appl Microbiol. 2020 May;70(5):342-348. | Inappropriate (epidemiological) study type and no intervention |
| Wei TZ, Wang H, Wu XQ, Lu Y, Guan SH, Dong FQ, Dong CL, Zhu GL, Bao YZ, Zhang J, Wang GY, Li HY. In Silico Screening of Potential Spike Glycoprotein Inhibitors of SARS-CoV-2 with Drug Repurposing Strategy. Chin J Integr Med. 2020 Sep;26(9):663-669. | Inappropriate (non-clinical) study type and intervention |
| Wen, C. C., et al. "Specific plant terpenoids and lignoids possess potent antiviral activities against severe acute respiratory syndrome coronavirus." J Med Chem 2007; 50(17): 4087-4095. | Inappropriate (non-clinical) study type and population (SARS) |
| Wen D, Shi Y, Zhang X, Lv G. Chinese medicine treatment of mastitis in COVID-19 patients: A protocol for systematic review. Medicine (Baltimore). 2020 Aug 28;99(35):e21656. | Not a primary study |
| Weng JK. Plant Solutions for the COVID-19 Pandemic and Beyond: Historical Reflections and Future Perspectives. Mol Plant. 2020 Jun 1;13(6):803-807. | Not a primary study |
| Wu, C., et al. "Heart injury signs are associated with higher and earlier mortality in coronavirus disease 2019 (COVID-19)." medRxiv: 2020.2002.2026.20028589. | Inappropriate (epidemiological) study type and no intervention |
| Wu, H. J., et al. "[Clinical observation on treatment of 40 SARS uncertain patients with integrative traditional Chinese and Western medicine]." Zhongguo Zhong Xi Yi Jie He Za Zhi 2003; 23(8): 572-574. | Inappropriate population (SARS) |
| Wu GS, Zhong J, Zheng NN, Wang CR, Jin HL, Ge GB, Han JY, Gao Y, Sheng LL, Zhang WD, Li HK. [Investigation of modulating effect of Qingfei Paidu Decoction on host metabolism and gut microbiome in rats]. Zhongguo Zhong Yao Za Zhi. 2020 Aug;45(15):3726-3739. Chinese.. | Inappropriate (non-clinical, animal) study type |
| Wu, Q., et al. "Epidemiological and Clinical Characteristics of Children with Coronavirus Disease 2019." medRxiv: 2020.2003.2019.20027078. | Inappropriate (epidemiological) study type and no intervention |
| Wyganowska-Swiatkowska M, Nohawica M, Grocholewicz K, Nowak G. Influence of Herbal Medicines on HMGB1 Release, SARS-CoV-2 Viral Attachment, Acute Respiratory Failure, and Sepsis. A Literature Review. Int J Mol Sci. 2020 Jun 30;21(13):4639. | Not a primary study |
| Xian Y, Zhang J, Bian Z, Zhou H, Zhang Z, Lin Z, Xu H. Bioactive natural compounds against human coronaviruses: a review and perspective. Acta Pharm Sin B. 2020 Jul;10(7):1163-1174. | Not a primary study |
| Xiao, P. G., et al. "[Some research clues on Chinese herbal medicine for SARS prevention and treatment]." Zhongguo Zhong Yao Za Zhi 2003; 28(6): 481-483. | Not a primary study |
| Xing, Y., et al. "Prolonged presence of SARS-CoV-2 in feces of pediatric patients during the convalescent phase." medRxiv: 2020.2003.2011.20033159. | Inappropriate (epidemiological) study type and no intervention |
| Xiong X, Wang P, Su K, Cho WC, Xing Y. Chinese herbal medicine for coronavirus disease 2019: A systematic review and meta-analysis. Pharmacol Res. 2020 Jul 2;160:105056. | Not a primary study |
| Xu, H., et al. "Acute Myocardial Injury of Patients with Coronavirus Disease 2019." medRxiv: 2020.2003.2005.20031591. | Inappropriate (epidemiological) study type and no intervention |
| Xu J, Zhang Y. Traditional Chinese Medicine treatment of COVID-19. Complement Ther Clin Pract. 2020 May;39:101165. | Not a primary study |
| Xu, K., et al. "[Management of corona virus disease-19 (COVID-19): the Zhejiang experience]." Zhejiang Da Xue Xue Bao Yi Xue Ban 49(1): 0. | Not a primary study |
| Xu, Z., et al. "Primary Care Practitioners' Response to 2019 Novel Coronavirus Outbreak in China." medRxiv: 2020.2002.2011.20022095. | Inappropriate (survey) study type and no intervention |
| Yan BH, et al. [Large- scale prospective clinical study on prophylactic intervention of COVID-19 in community population using Huoxiang Zhengqi Oral Liquid and Jinhao Jiere Granules]. Zhongguo Zhong Yao Za Zhi. 2020 Jul;45(13):2993-3000. Chinese. | Inappropriate intervention |
| Yan, D. X., et al. "[Discussion about treatment of severe acute respiratory syndrome based on syndrome differentiation]." Zhong Xi Yi Jie He Xue Bao 2004; 2(4): 241-244. | Not a primary study |
| Yan H, Zou Y, Zou C. [Mechanism of *QingfeiPaidu* decoction for treatment of COVID-19: analysis based on network pharmacology and molecular docking technology]. Nan Fang Yi Ke Da Xue Xue Bao. 2020 May 30;40(5):616-623. Chinese. | Inappropriate (non-clinical) study type |
| Yan J, Liu A, Huang J, Wu J, Fan H. Research Progress of Drug Treatment in Novel Coronavirus Pneumonia. AAPS PharmSciTech. 2020 May 13;21(4):130. | Not a primary study |
| Yan, S., et al. "Clinical Characteristics of Coronavirus Disease 2019 in Hainan, China." medRxiv: 2020.2003.2019.20038539. | Inappropriate (epidemiological) study type and no intervention |
| Yang, G., et al. "Angiotensin II Receptor Blockers and Angiotensin-Converting Enzyme Inhibitors Usage is Associated with Improved Inflammatory Status and Clinical Outcomes in COVID-19 Patients With Hypertension." medRxiv: 2020.2003.2031.20038935. | Inappropriate intervention (non-TCM) |
| Yang, G., et al. "Challenges and Countermeasures of Integrative Cancer Therapy in the Epidemic of COVID-19." Integr Cancer Ther 19: 1534735420912811. | Not a primary study |
| Yang KL, Gao Y, Yang FW, Liu M, Shi SZ, Chen YM, Zhang JH, Tian JH. [Analysis of traditional Chinese medicine from patent information sharing platform of coronavirus disease 2019 (COVID-19)]. Zhongguo Zhong Yao Za Zhi. 2020 Jul;45(13):3001-3006. Chinese. | Inappropriate (epidemiological) study type and no intervention |
| Yang M, Hu Z, Yue R. Efficacy and safety of Chinese herbal medicine for Coronavirus disease 2019: A protocol for systematic review and meta-analysis. Medicine (Baltimore). 2020 May 29;99(22):e20157. | Not a primary study |
| Yang, N., et al. "In-flight Transmission Cluster of COVID-19: A Retrospective Case Series." medRxiv: 2020.2003.2028.20040097. | Inappropriate (epidemiological) study type and no intervention |
| Yang, Y., et al. "Effect of continuous renal replacement therapy on all-cause mortality in COVID-19 patients undergoing invasive mechanical ventilation: a retrospective cohort study." medRxiv: 2020.2003.2016.20036780. | Inappropriate (epidemiological) study type and no intervention |
| Yang Q, Xie L, Zhang W, Zhao L, Wu H, Jiang J, Zou J, Liu J, Wu J, Chen Y, Wu J. Analysis of the clinical characteristics, drug treatments and prognoses of 136 patients with coronavirus disease 2019. J Clin Pharm Ther. 2020 Aug;45(4):609-616. doi: 10.1111/jcpt.13170. | Inappropriate (epidemiological) study type and no intervention |
| Yang R, Liu H, Bai C, Wang Y, Zhang X, Guo R, Wu S, Wang J, Leung E, Chang H, Li P, Liu T, Wang Y. Chemical composition and pharmacological mechanism of Qingfei Paidu Decoction and Ma Xing Shi Gan Decoction against Coronavirus Disease 2019 (COVID-19): In silico and experimental study. Pharmacol Res. 2020 Jul;157:104820. | Inappropriate (non-clinical) study type |
| Yang Y. Use of herbal drugs to treat COVID-19 should be with caution. Lancet. 2020 May 30;395(10238):1689-1690. | Not a primary study |
| Yao, Q., et al. “[Decoding herbal materials of representative TCM preparations with the multi-barcoding approach](http://biorxiv.org/content/early/2020/06/29/2020.06.29.177188)” bioRxiv 2020.06.29.177188 | Inappropriate (non-clinical) study type |
| Ye, Y.-a. "Guideline-based Chinese herbal medicine treatment plus standard care for severe coronavirus disease 2019 (G-CHAMPS): evidence from China." medRxiv: 2020.2003.2027.20044974. | Inappropriate intervention |
| Ye, Y.-a. "Guideline-based Chinese herbal medicine treatment plus standard care for severe coronavirus disease 2019 (G-CHAMPS): evidence from China. *Frontiers in Medicine* 7(256). | Inappropriate intervention |
| Yi, L., et al. "Small molecules blocking the entry of severe acute respiratory syndrome coronavirus into host cells." J Virol 2004; 78(20): 11334-11339. | Inappropriate (non-clinical) study type, population (SARS) and no intervention |
| Yin, J., et al. "In vitro and in vivo effects of Houttuynia cordata on infectious bronchitis virus." Avian Pathol 2011; 40(5): 491-498. | Inappropriate (non-clinical, animal) study type and intervention |
| Yu, H., et al. "The clinical and epidemiological features and hints of 82 confirmed COVID-19 pediatric cases aged 0-16 in Wuhan, China." medRxiv: 2020.2003.2015.20036319. | Inappropriate (epidemiological) study type and no intervention |
| Yu M, Zhang R, Ni P, Duan G. Chinese herbal medicine supplementation therapy on COVID-19. Pharmacol Res. 2020 Aug 29;160:105181. | Not a primary study |
| Yu, S., et al. "Network pharmacology-based analysis of the role of traditional Chinese herbal medicines in the treatment of COVID-19." Ann Palliat Med.2020 | Inappropriate (network pharmacology) study type |
| Yuan, J., et al. "Clinical Characteristics on 25 Discharged Patients with COVID-19 Virus Returning." medRxiv: 2020.2003.2006.20031377. | Inappropriate (epidemiological) study type and no intervention |
| Zeng M, Li L, Wu Z. Traditional Chinese medicine Lianhua Qingwen treating corona virus disease 2019(COVID-19): Meta-analysis of randomized controlled trials. PLoS One. 2020 Sep 11;15(9):e0238828. | Not a primary study |
| Zhang A, Li YP, Qiu M, Liu HB, Chen ZP, Wan P, Tao Y, Wang H, Wei DR, Li QT, Qin YL. [Impact of the Timing of Traditional Chinese Medicine Therapy on the Therapeutic Effect and Prognosis of Severe Coronavirus Disease 2019]. Zhongguo Yi Xue Ke Xue Yuan Xue Bao. 2020 Aug 30;42(4):521-530. Chinese. | Inappropriate (epidemiological) study type and no intervention |
| Zhang AH, Ren JL, Wang XJ. Reply to "The use of traditional Chinese medicines to treat SARS-CoV-2 may cause more harm than good". Pharmacol Res. 2020 Jul;157:104775. | Not a primary study |
| Zhang, B., et al. "Clinical characteristics of 82 death cases with COVID-19." medRxiv: 2020.2002.2026.20028191. | Inappropriate (epidemiological) study type and no intervention |
| Zhang C, Li J, Wu Z, Wang H, Que C, Zhao H, Wang G. Efficacy and safety of Anluohuaxian in the treatment of patients with severe Coronavirus disease 2019- a multicenter, open label, randomized controlled study: a structured summary of a study protocol for a randomised controlled trial. Trials. 2020 Jun 8;21(1):495. | Not a primary study and inappropriate intervention |
| Zhang, D., Zhang, X., Peng, B. *et al.* Network pharmacology suggests biochemical rationale for treating COVID-19 symptoms with a Traditional Chinese Medicine. *Commun Biol* 3**,**466 (2020) | Inappropriate (network pharmacology) study type |
| Zhang, D. H., et al. "In silico screening of Chinese herbal medicines with the potential to directly inhibit 2019 novel coronavirus." J Integr Med 2020; 18(2): 152-158. | Inappropriate (non-clinical) study type |
| Zhang D, Lyu JT, Zhang B, Zhang XM, Lin ZJ. [Pharmacovigilance study on drug-induced cardiac injury during treatment of COVID-19]. Zhongguo Zhong Yao Za Zhi. 2020 May;45(10):2275-2286. Chinese. | Inappropriate (non-clinical) study type |
| Zhang, h., et al. "Potential Factors for Prediction of Disease Severity of COVID-19 Patients." medRxiv: 2020.2003.2020.20039818. | Inappropriate (epidemiological) study type and no intervention |
| Zhang JL, Li WX, Li Y, Wong MS, Wang YJ, Zhang Y. Therapeutic options of TCM for organ injuries associated with COVID-19 and the underlying mechanism. Phytomedicine. 2020 Aug 5:153297. | Not a primary study |
| Zhang J, Xie B, Hashimoto K. Current status of potential therapeutic candidates for the COVID-19 crisis. Brain Behav Immun. 2020 Jul;87:59-73. | Not a primary study |
| Zhang Q, Wang Y, Qi C, Shen L, Li J. Clinical trial analysis of 2019-nCoV therapy registered in China. J Med Virol. 2020 Jun;92(6):540-545. | Not a primary study |
| Zhang Q, Cao F, Wang Y, Xu X, Sun Y, Li J, Qi X, Sun S, Ji G, Song B. The efficacy and safety of Jinhua Qinggan granule (JHQG) in the treatment of coronavirus disease 2019 (COVID-19): A protocol for systematic review and meta analysis. Medicine (Baltimore). 2020 Jun 12;99(24):e20531. | Not a primary study |
| Zhang Q, Cao F, Ji G, Xu X, Sun Y, Li J, Qi X, Sun S, Wang Y, Song B. The efficacy and safety of Lianhua Qingwen (LHQW) for coronavirus disease 2019 (COVID-19): A protocol for systematic review and meta analysis. Medicine (Baltimore). 2020 Jul 24;99(30):e20979. | Not a primary study |
| Zhang, R., et al. (2020). "Transmission and epidemiological characteristics of Severe Acute Respiratory Syndrome Coronavirus 2 (SARS-CoV-2) infected Pneumonia (COVID-19): preliminary evidence obtained in comparison with 2003-SARS." medRxiv: 2020.2001.2030.20019836. | Inappropriate (epidemiological) study type and no intervention |
| Zhang S, Zhu Q, Zhan C, Cheng W, Mingfang X, Fang M, Fang L. Acupressure therapy and Liu Zi Jue Qigong for pulmonary function and quality of life in patients with severe novel coronavirus pneumonia (COVID-19): a study protocol for a randomized controlled trial. Trials. 2020 Aug 27;21(1):751. | Not a primary study |
| Zhang W, Zhang P, Wang G, Cheng W, Chen J, Zhang X. Recent advances of therapeutic targets and potential drugs of COVID-19. Pharmazie. 2020 May 1;75(5):161-163. | Not a primary study |
| Zhang X, Cao D, Liu J, Zhang Q, Liu M. Efficacy and safety of Lianhua Qingwen combined with conventional antiviral Western Medicine in the treatment of coronavirus disease (covid-19) in 2019: Protocol for a systematic review and meta-analysis. Medicine (Baltimore). 2020 Jul 24;99(30):e21404. | Not a primary study |
| Zhang ZJ, et al. Active constituents and mechanisms of Respiratory Detox Shot, a traditional Chinese medicine prescription, for COVID-19 control and prevention: Network-molecular docking-LC-MS^E^ analysis. J Integr Med. 2020 May;18(3):229-241. | Inappropriate (non-clinical) type |
| Zhang Y, Xie H, Li Y, Li T, Yuan H, Fu X, Xie C. Qingfei Paidu decoction for treating COVID-19: A protocol for a meta-analysis and systematic review of randomized controlled trials. Medicine (Baltimore). 2020 Sep 4;99(36):e22040. | Not a primary study |
| Zhao HM, Xie YX, Wang C; Chinese Association of Rehabilitation Medicine; Respiratory Rehabilitation Committee of Chinese Association of Rehabilitation Medicine; Cardiopulmonary Rehabilitation Group of Chinese Society of Physical Medicine and Rehabilitation. Recommendations for respiratory rehabilitation in adults with coronavirus disease 2019. Chin Med J (Engl). 2020 Jul;133(13):1595-1602. | Not a primary study |
| Zhao, Y., et al. "Appealing for Efficient, Well Organized Clinical Trials on COVID-19." medRxiv: 2020.2003.2005.20031476. | Not a primary study |
| Zheng, Y., et al. "Study of the lymphocyte change between COVID-19 and non-COVID-19 pneumonia cases suggesting other factors besides uncontrolled inflammation contributed to multi-organ injury." medRxiv: 2020.2002.2019.20024885. | Inappropriate (non-clinical) type and intervention |
| Zhou, H., et al. "Urinalysis, but not blood biochemistry, detects the early renal-impairment in patients with COVID-19." medRxiv: 2020.2004.2003.20051722. | Inappropriate intervention (non-TCM, diagnostic) |
| Zhou H, Fang Y, Xu T, Ni WJ, Shen AZ, Meng XM. Potential therapeutic targets and promising drugs for combating SARS-CoV-2. Br J Pharmacol. 2020 Jul;177(14):3147-3161. doi: 10.1111/bph.15092. | Not a primary study |
| Zhou M, Liang Q, Pei Q, Xu F, Wen H. Chinese medicine for coronavirus disease 2019 as complementary therapy: A protocol for a systematic review and meta-analysis. Medicine (Baltimore). 2020 Aug 14;99(33):e21034. | Not a primary study |
| Zhou, X., et al. “Structure of SARS-CoV-2 main protease in the apo state reveals the inactive conformation” bioRxiv 2020.05.12.092171 | Inappropriate (non-clinical) type and nointervention |
| Zhu, R.-f., et al. "Systematic Review of the Registered Clinical Trials of Coronavirus Disease 2019 (COVID-19)." medRxiv: 2020.2003.2001.20029611. | Not a primary study |
| Zhuang, M., et al. "Procyanidins and butanol extract of Cinnamomi Cortex inhibit SARS-CoV infection." Antiviral Res 2009; 82(1): 73-81. | Inappropriate (non-clinical) study type, population (SARS) and intervention |
| Zhuang, Z., et al. "Estimation of local novel coronavirus (COVID-19) cases in Wuhan, China from off-site reported cases and population flow data from different sources." medRxiv: 2020.2003.2002.20030080. | Inappropriate (epidemiological) study type and no intervention |
| Zhuang W, Fan Z, Chu Y, Wang H, Yang Y, Wu L, Sun N, Sun G, Shen Y, Lin X, Guo G, Xi S. Chinese Patent Medicines in the Treatment of Coronavirus Disease 2019 (COVID-19) in China. Front Pharmacol. 2020 Jul 17;11:1066. | Inappropriate (survey) study type and no intervention |
| Zou J, Song DW, Niu JJ, Shi JW, Yang HL. Standardized out-patient diagnosis and treatment process for osteoporosis clinics during the COVID-19 pandemic. Eur Rev Med Pharmacol Sci. 2020 May;24(10):5778-5782. | Not a primary study |
| 常鹏程, 周志焕, 李艳伟, 张悦, 何涵宇, 于明坤, et al. 基于中医“未病先防”理论分析新型冠状病毒肺炎预防方案. 湖北中医杂志.1-6. | Not a primary study |
| 陈广, 刘艳娟, 涂胜豪, 陆付耳, 陈琢, 张明敏, et al. 新型冠状病毒感染肺炎的中医诊疗方案及预防方案. 内科急危重症杂志. 2020(01):6-8. | Not a primary study |
| 陈广, 刘艳娟. 华中科技大学同济医学院附属同济医院新型冠状病毒肺炎中医诊疗方案及预防方案. 医药导报. 2020(03):308-9. | Not a primary study |
| 陈慧君, 刘化冰. 利用专利大数据探索防治新型冠状病毒肺炎的中药材. 中国发明与专利. 2020;17(03):57-61. | Not a primary study |
| 陈婧, 王文清, 施春阳, 方建国. 新型冠状病毒肺炎（COVID-19）中医药防治的思考. 中草药. 2020;51(05):1106-12. | Not a primary study |
| 陈亚楠, 朱星昊, 苗明三, 孙旭, 刘怀民. 中医药防治新型冠状病毒肺炎的用药规律分析. 中国现代应用药学. 2020(05):542-8. | Not a primary study |
| 陈奕君, 王伟, 肖红斌. 基于液相色谱-质谱联用的清肺排毒汤中痕量马兜铃酸I的监测及定量分析. 药学学报.1-10. | Inappropriate (non-clinical) study type |
| 成颜琦, 陈希, 吴雨沁, 孙鼎, 喻晓, 李少滨, et al. 基于分型数据挖掘的中医药防治非危重型新型冠状病毒肺炎组方用药规律探析. 上海中医药杂志.1-8. | Inappropriate (data mining) study type |
| 程德忠, 李毅. 连花清瘟颗粒治疗54例新型冠状病毒肺炎患者临床分析及典型病例报道. 世界中医药. 2020;15(02):150-4. | Inappropriate (no comparasion) study type, |
| 戴敏, 肖阁敏, 王拥泽, 李林, 杨跃武, 谢和平, et al. 岭南新型冠状病毒肺炎临床表现的初步分析. 天津中医药. 2020(02):132-6. | Inappropriate (non-clinical) study type |
| 单卓程, 叶人, 袁拯忠, 朱文宗, 刘刚, 朱小区, et al. 温州地区中西医结合治疗新型冠状病毒肺炎体会. 新中医. 2020:1-7. | Inappropriate (non-clinical) study type, |
| 丁瑞丛, 龙清华, 刘玲, 王平, 黄晓宇, 明淑萍. 运用达原饮治疗新型冠状病毒肺炎的体会. 中医杂志. 2020:1-5. | Not a primary study |
| 范建新, 秦雪梅, 李震宇. 基于网络药理学和分子对接技术的款冬花在清肺排毒汤治疗新型冠状病毒肺炎（COVID-19）中的作用分析. 中草药.1-9. | Inappropriate (network pharmacology) study type |
| 范丽洁, 杜惠兰, 段梅. 妊娠期新型冠状病毒肺炎的中医认识及辨证与治疗要点. 中医杂志.1-5. | Not a primary study |
| 范天田, 陈永灿, 白钰, 马凤岐, 王恒苍, 杨益萍, et al. 2019冠状病毒病（COVID-19）基于推荐诊疗方案的中医用药特点分析. 浙江大学学报(医学版).1-17. | Not a primary study |
| 龚普阳, 郭瑜婕, 李晓朋, 王楠, 顾健. 基于网络药理学与分子对接技术的金花清感颗粒防治新型冠状病毒肺炎的潜在药效物质研究. 中草药.1-9. | Inappropriate (network pharmacology) study type |
| 龚新月, 魏大荣, 龚雪, 熊燕, 汪谭, 牟方政. 80例新型冠状病毒肺炎患者中医临床特征及证候分析. 中国中医药信息杂志. 2020:1-8. | Inappropriate (non-clinical) type |
| 龚雪, 牟方政, 魏大荣, 窦露, 龚新月, 汪潭, et al. 225例新型冠状病毒肺炎的临床特征及中医药应用分析. 世界中医药. 2020:1-13. | Inappropriate (non-clinical) study type |
| 顾植山. 五运六气看当前新型冠状病毒肺炎疫情. 世界中医药. 2020;15(02):144-9. | Not a primary study |
| 何钦, 叶旭星, 徐斌. 中西医结合治疗新型冠状病毒肺炎验案2则. 中国中西医结合杂志. 2020(03):378-9. | Inappropriate (case reportl) study type |
| 何天目, 段灿灿, 李晓飞, 张建永. 基于网络药理学和分子对接探索血必净注射液治疗冠状病毒肺炎的潜在机制. 中国现代应用药学. 2020;37(04):398-405. | Inappropriate (network pharmacology) study type |
| 贺煜竣, 邢博文, 杨凌毓, 宋伯骐, 张亚兰, 刘未艾. 从膜原理论探讨达原饮对新型冠状病毒肺炎早期的治疗. 世界科学技术-中医药现代化.1-5. | Not a primary study |
| 洪炳杰, 陈晓彤. 新型冠病毒肺炎各地中医药诊疗方案的中医证素分布与遣方用药规律分析. 辽宁中医杂志.1-6. | Not a primary study |
| 胡美霖, 董若兰, 陈广, 董慧, 张明敏, 陆付耳, et al. 中西医结合治疗重症新型冠状病毒肺炎临床病例1例. 中国中西医结合杂志. 2020(02):228-30. | Inappropriate (case reportl) study type |
| 胡雯婷, 戴红. 基于病证结合角度探析新型冠状病毒肺炎的中西医结合诊疗模式. 中医学报. 2020;35(03):501-3. | Not a primary study |
| 胡晓燕, 张仕瑾, 杨思芸, 郭廷东, 黄梅, 苏强, et al. 治疗新型冠状病毒肺炎的中药联用常用西药不良相互作用分析. 中国药业. 2020;29(06):29-32. | Inappropriate (non-clinical) study type and no intervention |
| 霍宣东, 先小乐. 中医学关于新型冠状病毒肺炎的几点认识与思考. 中国民间疗法. 2020(06):4-6. | Not a primary study |
| 吉米丽汗·司马依, 买买提明·努尔买买提, 艾尼瓦尔·吾买尔, 买尔旦·玉苏甫, 木哈待斯·努尔, 努丽比亚·买合木提, et al. 基于网络药理学及分子对接探索金花清感颗粒辅助治疗新型冠状病毒肺炎（COVID-19）活性成分研究. 中药材.1-10. | Inappropriate (network pharmacology) study type |
| 贾振华, 李红蓉, 常丽萍, 魏聪. 中医学应对疫病的历史回顾与思考. 中国实验方剂学杂志.1-6. | Not a primary study |
| 贾振华. 网络病理论指导新型冠状病毒肺 | Inappropriate (network theory) study type |
| 蒋鹏飞, 李书楠, 刘培, 彭俊, 彭清华. 全国各地区新型冠状病毒肺炎中医防治方案分析. 中医学报.1-20. | Not a primary study |
| 蒋谦谦, 陈新宇. 基于方证辨证对清肺排毒汤速效治疗新型冠状病毒肺炎的思考. 中医杂志.1-3. | Not a primary study |
| 靳英辉, 蔡林, 程真顺, 程虹, 邓通, 范逸品, et al. 新型冠状病毒(2019-nCoV)感染的肺炎诊疗快速建议指南(标准版). 解放军医学杂志. 2020;45(01):1-20. | Inappropriate (official guidance) study type |
| 康乐, 苗晋鑫, 苗明三, 张亚楠, 张瑾. 血必净类制剂治疗新冠病毒肺炎的作用特点. 中药药理与临床.1-10. | Inappropriate (network pharmacology) study type |
| 孔艺, 林莉莉, 陈永, 赖莎, 吴红卫, 陈吉生. 基于网络药理学探讨血必净注射液治疗新型冠状病毒肺炎机制. 世界科学技术-中医药现代化.1-9. | Inappropriate (pharmacology) study type |
| 李承羽, 张晓雨, 刘斯, 商洪才. 血必净注射液治疗新型冠状病毒感染的肺炎(COVID-19)证据基础及研究前瞻. 世界科学技术-中医药现代化.1-6. | Inappropriate (pharmacology) study type |
| 李春波, 苏韫, 刘永琦, 薛轩, 龚红霞, 李婷婷, et al. 清肺排毒汤治疗新型冠状病毒肺炎的中医理论及现代药理学机制探讨. 中医杂志.1-4. | Inappropriate (pharmacology) study type |
| 李红蓉, 常丽萍, 魏聪, 贾振华. 连花清瘟治疗新型冠状病毒肺炎的理论研究基础和临床疗效. 世界中医药.1-5. | Inappropriate (non-clinical) study type |
| 李慧, 李闻涓, 侯宁宁, 陈颖, 罗兴洪. 中医在瘟疫防治中的作用. 中国合理用药探索. 2020;17(02):14-20. | Inappropriate (non-clinical) study type and no intervention |
| 李坚, E.Olaleye O, 余玄, 贾伟伟, 杨军令, 吕闯, et al. 血必净注射液联合抗生素治疗脓毒症：两类药物间高水平药代和谐. 中国临床药理学与治疗学.1-3. | Inappropriate type for pyohemia |
| 李婧, 马小兵, 沈杰, 张志锋. 基于文献挖掘与分子对接技术的抗新型冠状病毒中药活性成分筛选. 中草药. 2020(04):845-50. | Inappropriate (data mining) study type |
| 李思聪, 冯祥, 毕磊, 刘欣. 新型冠状病毒肺炎诊疗方案中成药选用分析与药理研究进展. 中药材. 2020(03):764-71. | Inappropriate (pharmacology) study type |
| 李闻涓, 任晋生, 李慧, 陈颖, 侯宁宁, 罗兴洪. 转化医学在抗击新型冠状病毒中的应用. 药物评价研究. 2020;43(03):392-5. | Inappropriate (non-clinical) study type and intervention |
| 李西亮, 孙红兵, 宋言壮, 卜利伟, 钟坚娥, 孙学全. 孙学全通腑益气化瘀法治疗血管狭窄临证心悟. 江苏中医药. 2020(02):21-4. | Inappropriate (non-clinical) study type and intervention for narrow blood tube |
| 李希, 蔡艳萍, 涂思义, 巫雅婷, 李丽君. 从中医辨证论治分期治疗新型冠状病毒肺炎. 福建中医药. 2020;51(01):8-10. | Inappropriate (non-clinical) study type and no intervention |
| 李修洋, 宋斌, 雷烨, 李青伟, 郑玉娇, 刘文科, et al. 《新型冠状病毒肺炎诊疗方案（试行第六版）》中医诊疗方案解读. 吉林中医药.1-8. | Not a primary study |
| 凌晓颖, 陶嘉磊, 孙逊, 袁斌. 基于网络药理学探讨连花清瘟方抗冠状病毒的物质基础及机制研究. 中草药.1-8. | Inappropriate (network pharmacology) study type |
| 刘昌孝, 王玉丽, 张洪兵, 田成旺, 黄鹤, 张铁军. 基于新型冠状病毒感染防控需求，重视中药科学研发与应用. 中草药. 2020;51(06):1361-74. | Not a primary study and no intervention |
| 刘菊, 崔瑛, 白明学, 张红伟, 金云隆, 吕鹏. 基于中医药防治新型冠状病毒肺炎的用药探析. 中草药. 2020;51(04):860-5. | Not a primary study |
| 刘闰平, 葛俊德, 钟颖, 郑淇, 孙蓉. 基于干预细胞因子风暴文献挖掘的中医药治疗重症新型冠状病毒肺炎探讨. 中草药. 2020;51(05):1096-105. | Inappropriate (data mining) study type |
| 刘颖, 刘丽, 曹苗苗, 温剑, 周燊, 江华, et al. 《新型冠状病毒肺炎诊疗方案（试行第六版）》中医学观察期患者的中成药治疗文献分析. 临床药物治疗杂志. 2020;18(02):62-6. | Not a primary study |
| 刘媛, 胡秋红, 黄柏学, 司亚玲, 陈四清. 从伏气温病探讨新型冠状病毒肺炎的“有病无症”. 中医学报.1-7. | Not a primary study |
| 陆建荣. 531例流感样疾病病毒病原学与中医证候相关性分析 [硕士]: 广州中医药大学; 2012. | Inappropriate population (H1N1) |
| 鹿振辉, 邱磊, 张少言, 江峰, 张惠勇, 肖臻, et al. 试论新型冠状病毒肺炎重症“寒湿水饮闭肺，命门之火不振”之变. 中国中医药信息杂志.1-3. | Inappropriate (pharmacology) study type |
| 罗蒙, 江波, 徐鸿婕, 杨倩, 周雪情, 吕琨, et al. 新型冠状病毒肺炎患者死亡影响因素分析. 中草药. 2020;51(06):1450-4. | Not a primary study |
| 吕文亮. 基于《湖北省新型冠状病毒肺炎中医药防治指引(试行)》的解读. 世界中医药. 2020;15(02):125-8. | Not a primary study |
| 马婧, 霍晓乾, 陈茜, 朱卫星, 姚美村, 乔延江, et al. 基于Mpro和PLP筛选潜在抗新型冠状病毒中药研究. 中国中药杂志.1-8. | Inappropriate (Mprd and PLP screening ) study type |
| 马青云, 刘辰, 杜海涛, 张贵晟, 闫滨, 孙启慧, et al. 基于高通量分子对接虚拟筛选SARS-CoV-2 3CL水解酶中药小分子抑制剂及抗新型冠状病毒肺炎（COVID-19）的中药及其复方预测. 中草药. 2020;51(06):1397-405. | Inappropriate (non-clinical) study type and no intervention |
| 马战平, 路波, 王志梅, 杨栓柱. 中研益肺解毒汤防治新型冠状病毒肺炎临床观察. 陕西中医. 2020(04):424-6. | Inappropriate intervetion |
| 马战平, 阴智敏, 魏耕树, 白丽君, 李猛, 刘素香, et al. 陕西省新型冠状病毒感染的肺炎中医药治疗方案(试行第二版). 陕西中医. 2020;41(03):275-7. | Not a primary study |
| 毛昀, 苏毅馨, 薛鹏, 李林潞, 朱世杰. 金花清感颗粒治疗新型冠状病毒肺炎作用机制探讨. 中药材.1-8. | Not a primary study |
| 南征, 王檀, 仕丽, 方雅堃, 鲍鹏杰, 祝志岳, et al. 吉林省新型冠状病毒肺炎中医诊治思路与方法. 吉林中医药. 2020;40(02):141-4. | Not a primary study |
| 庞文渊, 王乔羽, 赵志刚. 新型冠状病毒肺炎的药物治疗方案及研究进展. 临床药物治疗杂志. 2020;18(02):24-9. | Not a primary study |
| 漆国栋, 漆伟, 江琼, 申开琴, 张雪, 张练. 连花清瘟结合西医方案对新冠肺炎普通型患者疗效的系统评价. 中医药临床杂志.1-9. | Not a primary study |
| 秦裕辉, 胡方林, 葛金文. 新冠系列方预防新型冠状病毒感染用方探讨. 湖南中医药大学学报. 2020(02):129-33. | Not a primary study |
| 任秀华, 祁星星, 左琴, 汤杰, 刘东. 方舱医院813例新型冠状病毒肺炎患者治疗用药分析. 医药导报.1-10. | Not a primary study |
| 任越, 姚美村, 霍晓乾, 谷宇, 朱卫星, 乔延江, et al. 抗新型冠状病毒方剂基于花生四烯酸代谢通路防治“细胞因子风暴”的研究. 中国中药杂志.1-8. | Inappropriate intervention (non-TCM) |
| 阮小风, 冯雨薇, 赵康, 黄橘村, 陈雨, 刘黎明. 从湿温论治重型新型冠状病毒肺炎老年患者1例. 上海中医药杂志.1-4. | Inappropriate (case reportl) study type |
| 尚晓娟, 刘海军, 朱丽红, 何梅梅, 辛克锋. 安徽省阜阳市新型冠状病毒肺炎患者的流行病学及临床特征分析. 疑难病杂志. 2020:1-5. | Inappropriate (case reportl) study type |
| 邵仲柏, 朱月霞, 刘书豪, 蒋凯俊, 吴琦, 沈金阳, et al. 临床使用治疗新型冠状病毒肺炎中药复方中高频数中药抗病毒研究概述. 中草药. 2020(05):1153-8. | Not a primary study |
| 沈浮, 付中应, 吴泳蓉, 李玲, 赵昱东, 夏雨, et al. 基于网络药理学及高通量分子对接研究金花清感颗粒中结合SARS-CoV-2特定靶蛋白的活性化合物干预COVID-19的潜在分子机制. 世界科学技术-中医药现代化.1-10. | Inappropriate (network pharmacology) study type |
| 施烜, 魏娟, 刘美云, 金小红, 周焕平, 朱万莉, et al. 血必净注射液治疗新型冠状病毒肺炎的整体调控作用研究. 上海中医药杂志.1-7. | Not a primary study |
| 史光伟, 梁永林, 苏颖. 新型冠状病毒肺炎太阳少阳太阴病脉证并治. 甘肃中医药大学学报. 2020;37(01):23-7. | Inappropriate (pharmacology) study type |
| 孙梦华, 谢雁鸣. 针对目前已注册的上市中成药治疗新型冠状病毒肺炎临床研究分析. 世界科学技术-中医药现代化.1-10. | Inappropriate (non-clinical) study type |
| 田朝晖, 向建军, 葛舰, 秦开烈, 李玉英, 王奎, et al. 清肺排毒汤治疗新型冠状病毒肺炎理论分析与临床实践. 世界中医药. 2020:1-5. | Inappropriate (pharmacology) study type |
| 田野, 李瑞明, 任红微, 李德坤, 鞠爱春, 何毅, et al. 生脉散用于新型冠状病毒肺炎恢复期治疗的可行性探讨. 药物评价研究. 2020(03):378-83. | Inappropriate (pharmacology) study type |
| 汪升早, 王华军, 陈鸿明, 岳玉, 卜凡进, 张雪梅. 连花清瘟胶囊和α-干扰素联合洛匹那韦/利托那韦治疗新型冠状病毒肺炎30例. 蚌埠医学院学报. 2020;45(02):154-5. | Inappropriate intervention (non-TCM) |
| 王传池, 吴珊, 江丽杰, 许伟明, 杨燕, 胡镜清. 全国各地区新型冠状病毒肺炎中医药诊治方案综合分析. 世界科学技术-中医药现代化.1-7. | Not a primary study |
| 王法财, 沈炳香, 何春远, 赵为陈, 聂松柳. 连花清瘟颗粒对新型冠状病毒肺炎的临床疗效及其机制的网络药理学研究. 中药药理与临床. 2020:1-22. | Inappropriate (network pharmacology) study type |
| 王刚, 金劲松. 新型冠状病毒肺炎病机演变规律及经方的治疗实践——附验案4则. 江苏中医药. 2020:1-5. | Inappropriate (case reportl) study type |
| 王辉, 金鑫瑶, 庞博, 刘春香, 郑文科, 杨丰文, et al. 中医药干预新型冠状病毒肺炎临床研究方案分析. 中国中药杂志. 2020:1-11. | Not a primary study |
| 王金榜, 梁保丽, 孙树椿. 新型冠状病毒(COVID-19)感染性肺炎现代中医诊疗建议方案与探讨. 世界中医药. 2020(01):35-46. | Not a primary study |
| 王林, 杨志华, 张浩然, 于航星, 杨康, 付宝慧, et al. 连花清瘟治疗新型冠状病毒(2019-nCoV)肺炎网络药理学研究与初证. 中药材. 2020(03):772-8. | Inappropriate (network pharmacology) study type |
| 王琳, 王静, 涂杰霞, 司果, 姜琪, 任雪松, et al. 新型冠状病毒肺炎患者的治疗及不良反应监护. 中国现代应用药学. 2020;37(05):530-5. | Inappropriate (pro-diagnosis) study type |
| 王宁 本王. 专家详解第六版诊疗方案.  2020-02-21. | Not a primary study |
| 王怡菲, 邱模炎, 裴颢, 闫二萍, 张琼英, 刘淑娟, et al. 中医药辨治新型冠状病毒肺炎的组方及用药规律探析. 世界中医药.1-4. | Inappropriate (pharmacology) study type |
| 王玉光, 齐文升, 马家驹, 阮连国, 卢幼然, 李旭成, et al. 新型冠状病毒肺炎中医临床特征与辨证治疗初探. 中医杂志. 2020(04):281-5. | Not a primary study |
| 魏朝法, 杜佳, 翟煦, 李佳佳, 张佳乐, 王柳青. 细辛在防治新型冠状病毒肺炎方剂中的应用安全性思考. 医学争鸣.1-10. | Inappropriate (non-clinical) study type and intervention |
| 魏华民, 李杨帆, 俞静, 林海. 从中医学角度浅析新型冠状病毒肺炎愈后遗症防控. 世界中医药. 2020;15(02):166-71. | Inappropriate (non-clinical) study type and intervention |
| 吴昊, 王佳琪, 杨雨薇, 李天怡, 曹一佳, 曲玉霞, et al. 基于网络药理学和分子对接技术初步探索“清肺排毒汤”抗新型冠状病毒肺炎作用机制. 药学学报. 2020;55(03):374-83. | Inappropriate (network pharmacology) study type |
| 吴伟, 温敏勇, 詹少锋, 刘小斌, 黄衍寿, 冼绍祥, et al. 基于中医瘟疫火热病机探讨新型冠状病毒肺炎辨证论治. 中国中西医结合杂志. 2020;40(03):272-4. | Inappropriate (pharmacology) study type |
| 熊微, 冉京燕, 谢雪佳, 夏亿红, 兰标, 汪梦蝶, et al. 治疗新型冠状病毒肺炎中成药的药理作用与临床应用. 医药导报.1-26. | Inappropriate (pharmacology) study type |
| 徐天馥, 贺成功, 杨坤. 基于网络药理学清肺排毒汤治疗新冠肺炎的物质基础及作用机制研究. 天然产物研究与开发.1-12. | Inappropriate (network pharmacology) study type |
| 徐旭, 张莹, 李新, 李晓霞. 各地区中医药预防新型冠状病毒肺炎(COVID-19)方案分析. 中草药. 2020(04):866-72. | Not a primary study |
| 许二平, 徐江雁, 徐立然, 魏征, 孟鹏飞, 宋夕元, et al. 新型冠状病毒肺炎常见病症经方辨治纲要. 中医学报.1-6. | Inappropriate (pharmacology) study type and no intervention |
| 许禄华, 李彦荣, 郑丹如, 邵宗钫, 闻思齐, 林丰夏, et al. 基于“因时制宜”探讨新型冠状病毒肺炎不同阶段的处方用药规律. 中国实验方剂学杂志.1-11. | Not a primary study |
| 薛伯寿, 姚魁武, 薛燕星. “清肺排毒汤”快速有效治疗新型冠状病毒肺炎的中医理论分析. 中医杂志. 2020;61(06):461-2. | Inappropriate (pharmacology) study type |
| 杨波, 卢欣, 于睿莉, 张皓旻, 张钧栋, 迟小华, et al. 冠状病毒感染相关多器官功能障碍综合征分子机制和干预药物的生物信息学预测及其对新型冠状病毒肺炎的意义. 中华老年多器官疾病杂志. 2020(03):182-6. | Inappropriate (non-clinical) study type and no intervention |
| 杨羽君, 鄂秀辉, 任红微, 何毅, 周水平. 中医药治疗人类高致病性冠状病毒SARS-CoV-2与SARS-CoV感染肺炎的思考. 中草药. 2020(06):1427-34. | Not a primary study |
| 尹明星, 曹艳, 施春阳, 王文清, 方建国. 中药防治细胞因子风暴的研究进展. 中草药. 2020;51(05):1089-95. | Not a primary study |
| 尹相乾, 姚博, 马文辉. 基于三部六病理论体系为基层医疗机构提供防治新型冠状病毒肺炎的思路. 世界中医药. 2020(02):177-80. | Inappropriate (pharmacology) study type |
| 喻培勋, 姬晓花, 刘静, 王刚. 六经辨证应用于COVID-19探讨. 河南中医.1-2. | Inappropriate (pharmacology) study type |
| 袁蓉, 信琪琪, 唐仕欢, 丛伟红. 中医整体观指导下的COVID-19治疗——同时着眼于病毒和宿主的疗法. 中国中药杂志.1-6. | Not a primary study |
| 岳萍, 唐仕欢, 于欢, 王芳, 赖昕, 吴金鹏, et al. 新型冠状病毒肺炎中医防治方案的病机与组方规律分析. 中国实验方剂学杂志.1-9. | Not a primary study |
| 张传雷, 李彬, 李纪高, 邹善思, 董郑霞, 王新亭. 从中医瘟病理论辨证论治新型冠状病毒肺炎. 中医学报. 2020(03):494-7. | Inappropriate (pharmacology) study type |
| 张从玉, 张帅, 王婉, 姜雪强. 血必净治疗新型冠状病毒肺炎的临床疗效观察. 中国医院药学杂志.1-5. | Inappropriate (no comparasion) study type, |
| 张宏亮, 黄振光, 蒋霞, 丘岳, 黄天敏, 林忠秋, et al. 基于网络药理学研究麻杏石甘汤治疗重型新冠肺炎的作用机制. 中药材.1-8. | Inappropriate (network pharmacology) study type |
| 张吉仲, 降拥彭措, 纳顺达来, 库尔班·艾力, 李凤珍, 王孝蓉, et al. 民族医药对新型冠状病毒肺炎的认识及防治措施. 中草药. 2020;51(06):1463-75. | Inappropriate (non-clinical) study type and no intervention |
| 张荣珍, 杨元元, 胡军平. 从疫病护阳的重要性解读清肺排毒汤方义. 中医药临床杂志.1-7. | Inappropriate (pharmacology) study type |
| 张巍岚, 王相东, 王郁金, 谭从娥. 从国医大师邓铁涛治“非典”经验探讨新型冠状病毒肺炎中医诊疗思路. 中医学报. 2020(03):483-6. | Inappropriate (non-clinical) study type and no intervention |
| 张馨月, 郑佳昆. 从戾气学说谈新型冠状病毒肺炎的中医诊治. 河南中医. 2020(03):336-8. | Inappropriate (pharmacology) study type |
| 张岩, 唐德志, 舒冰, 李文雄, 张佳莉, 李钺, et al. 基于文献探讨中药干预新型冠状病毒肺炎作用机制. 中医杂志.1-8. | Not a primary study |
| 张彦丽, 赵薇, 靳梦亚, 桂月, 张谦, 宫朝玲, et al. 中医药防治新型冠状病毒肺炎的用药监护. 中国药业. 2020;29(05):27-34. | Inappropriate (non-clinical) study type |
| 张依倩, 史嘉雯, 刘园华, 周浩, 蔡晓庆, 温晋, et al. 补气活血中药在新型冠状病毒肺炎低氧血症的潜在临床应用价值及其机制浅析. 中草药. 2020(06):1435-42. | Inappropriate (network pharmacology) study type |
| 张治国, 程引, 黄煌. 基于“药人”学说的新型冠状病毒肺炎预防方药探讨. 中国民间疗法. 2020(06):1-4. | Inappropriate (pharmacology) study type |
| 赵东凯, 蔡鸿彦, 罗威, 黄颖新, 马翮, 陈亦洋, et al. 中西医结合治疗新型冠状病毒肺炎1例. 吉林中医药. 2020:1-4. | Inappropriate (case reportl) study type |
| 赵静, 田赛赛, 杨健, 刘剑锋, 张卫东. 清肺排毒汤治疗新型冠状病毒肺炎机制的网络药理学探讨. 中草药. 2020;51(04):829-35. | Inappropriate (network pharmacology) study type |
| 赵亮. 浅析经方对新型冠状病毒肺炎的辨治思路. 中国民间疗法. 2020(05):1-2+8. | Inappropriate (pharmacology) study type |
| 郑文科, 张俊华, 杨丰文, 王玉光, 刘清泉, 张伯礼. 中医药防治新型冠状病毒肺炎各地诊疗方案综合分析. 中医杂志. 2020(04):277-80. | Not a primary study |
| 周洪立, 陈海彬, 周红光, 邱雯莉, 李沐涵, 石海波, et al. 中医“治未病”思想在防控新型冠状病毒肺炎中的应用. 河南中医. 2020(03):332-5. | Inappropriate (pharmacology) study type |
| 周铭心, 王苗. 新型冠状病毒肺炎中医防治策略与方药筛选. 中医学报. 2020(03):458-63. | Not a primary study |
| 周严严, 高文雅, 顾欣如, 陈周全, 赵海誉, 边宝林, et al. 基于UHPLC-LTQ-Orbitrap-MS技术的清肺排毒汤化学成分鉴定及归属研究. 中国中药杂志.1-15. | Inappropriate (UHPLC-LTQ-Orbitrap-MS) study type |
| 朱亮, 徐菲拉, 王群星, 徐斌. 浙江省金华市防治新型冠状病毒感染肺炎中药处方分析. 浙江中西医结合杂志. 2020(03):179-81. | Not a primary study |
| 宗阳, 丁美林, 贾可可, 马世堂, 居文政. 基于网络药理学和分子对接法探寻达原饮治疗新型冠状病毒(2019-nCoV)肺炎活性化合物的研究. 中草药.1-9. | Inappropriate (network pharmacology) study type |
| 何天目, 段灿灿, 李晓飞, 张建永. 基于网络药理学和分子对接探索血必净注射液治疗冠状病毒肺炎的潜在机制. 中国现代应用药学. 2020;37(04):398-405. | Network pharmacology and macromolecular docking |
| 何清湖, 刘应科, 孙相如, 孙英凯, 孙光荣. 国家卫生健康委员会、国家中医药管理局推荐使用“清肺排毒汤”的意义与作用. 中医杂志. 2020;61(10):829-32. | Non-clinical research |
| 凌晓颖, 陶嘉磊, 孙逊, 袁斌. 基于网络药理学的连花清瘟方抗冠状病毒的物质基础及机制探讨. 中草药. 2020;51(07):1723-30. | Network pharmacology |
| 刘厚利, 呼兴华, 何莉, 刘双, 许建秦. 清肺排毒汤组方原理、药物用量及煎煮方法概述. 陕西中医. 2020;41(05):560-2. | Non-clinic research |
| 刘浩, 高原, 尹小星, 杨阳, 曲妮妮. 浅析清肺排毒汤治疗新型冠状病毒肺炎. 辽宁中医药大学学报.1-9. | Non-clinic research (CM theory) |
| 刘源, 刘金豹, 彭伟. 基于网络药理学探讨化湿败毒方治疗新型冠状病毒肺炎(COVID-19)的作用机制. 海南医学院学报. 2020;26(11):804-13. | Network pharmacology |
| 刘颖, 刘丽, 曹苗苗, 温剑, 周燊, 江华, et al. 《新型冠状病毒肺炎诊疗方案（试行第六版）》中医学观察期患者的中成药治疗文献分析. 临床药物治疗杂志. 2020;18(02):62-6. | Literature review |
| 吉米丽汗·司马依, 买买提明·努尔买买提, 艾尼瓦尔·吾买尔, 买尔旦·玉苏甫, 卡依赛尔·阿布都肉苏力, 木哈待斯·努尔, et al. 基于网络药理学的血必净注射液干预细胞因子风暴辅助治疗重症新型冠状病毒肺炎的潜在机制研究. 天然产物研究与开发. 2020;32(08):1316-23. | Network pharmacology |
| 吉米丽汗·司马依, 买买提明·努尔买买提, 艾尼瓦尔·吾买尔, 买尔旦·玉苏甫, 木哈待斯·努尔, 努丽比亚·买合木提, et al. 基于网络药理学及分子对接探索金花清感颗粒辅助治疗新型冠状病毒肺炎（COVID-19）活性成分研究. 中药材. 2020(05):1275-83. | Network pharmacology |
| 吴昊, 王佳琪, 杨雨薇, 李天怡, 曹一佳, 曲玉霞, et al. 基于网络药理学和分子对接技术初步探索“清肺排毒汤”抗新型冠状病毒肺炎作用机制. 药学学报. 2020;55(03):374-83. | Network pharmacology and macromolecular docking |
| 吴高松, 钟婧, 郑宁宁, 王超然, 金红利, 葛广波, et al. 清肺排毒汤对大鼠整体代谢及肠道菌群的调节作用研究. 中国中药杂志. 2020;45(15):3726-39. | Animal research |
| 周宝宽. 清肺排毒汤治疗新型冠状病毒肺炎机制解析. 辽宁中医药大学学报. 2020;22(07):13-9. | Non-clinic research |
| 周梦琪, 杨璐平, 马浩洁, 程翠翠, 张宇昕, 张锦坤, et al. 清肺排毒汤干预新冠肺炎细胞因子风暴机制的网络药理学研究. 海南医学院学报. 2020;26(10):721-9. | Network pharmacology |
| 姚佳, 史晓燕, 陈秋, 凡思敏, 杨仁旭, 彭波. 清肺排毒汤治疗新型冠状病毒肺炎的方药理论研究. 辽宁中医杂志. 2020;47(05):94-8. | Non-clinic research |
| 孔艺, 林莉莉, 陈永, 赖莎, 吴红卫, 陈吉生. 基于网络药理学探讨血必净注射液治疗新型冠状病毒肺炎机制. 世界科学技术-中医药现代化. 2020;22(03):552-60. | Network pharmacology |
| 孙光荣. 中医药向新冠肺炎“亮剑”——国家卫生健康委、国家中医药局推荐使用“清肺排毒汤”的意义和作用. 健康中国观察. 2020(04):57-60. | Non-clinic research |
| 孙逊, 陶嘉磊, 许少菊, 袁斌. 基于网络药理学探究化湿败毒方治疗新型冠状病毒肺炎的分子机制. 中药材. 2020(08):2050-5. | Network pharmacology and macromolecular docking |
| 孟军华, 何阳, 陈茜, 高强, 陈永刚, 安靖. 清肺排毒汤治疗普通型/重型新型冠状病毒肺炎的回顾性研究. 中国医院药学杂志.1-7. | Non-comparison medication |
| 宋江秀, 张忠会, 赵帅眉, 李慧, 郭洁. 新型冠状病毒肺炎（COVID-19）防治用中药专利信息研报. 药物评价研究. 2020;43(04):565-90. | Non-clinic research |
| 康乐, 苗晋鑫, 苗明三, 张亚楠, 张瑾. 血必净类制剂治疗新冠病毒肺炎的作用特点. 中药药理与临床. 2020;36(02):11-5. | Literature review |
| 廖垚, 殷贝, 金镇, 鲍广兵, 李佑生. 化湿败毒方治疗重型新型冠状病毒肺炎的中医理论分析及现代药理学机制探讨. 海南医学院学报. 2020;26(16):1209-13. | Non-clinic research (CM theory) |
| 张丽娟, 范恒, 陈瑞, 朱习文, 王文竹, 崔丹丹, et al. 从临床实践谈清肺排毒汤的合理应用. 中医杂志.1-5. | Non-clinic research |
| 张依梦, 耿立梅, 陈杰勇, 马蕴蕾, 郑强, 郭登洲. 清肺排毒汤联合西药治愈新型冠状病毒肺炎重型病案1例. 天津中医药. 2020;37(08):861-5. | Case study |
| 张嘉俊, 李珊珊, 李江娅, 张欣月, 林浩畅, 杨雅清, et al. 连花清瘟结合西医治疗新型冠状病毒肺炎患者的文献分析. 中国医院药学杂志.1-5. | Literature review |
| 张文斌, 刘利男, 王震, 刘杨. 连花清瘟联合西医治疗新冠肺炎普通型患者疗效及安全性meta分析. 海南医学院学报. 2020;26(14):1045-50. | Meta analysis |
| 张瑞雪, 张彩云, 袁亚美, 方正清, 王茜, 朱林. “易阳通元”防治新冠肺炎作用探讨. 现代中医药. 2020;40(03):1-6. | Non-clinic research (CM theory) |
| 张荣珍, 杨元元, 胡军平. 从疫病护阳的重要性解读清肺排毒汤方义. 中医药临床杂志. 2020;32(05):825-8. | Non-clinic research (CM theory) |
| 张雯雯, 柳越冬, 陈萌, 张斯瑶, 高泉, 冀文鹏, et al. 清肺排毒汤再识. 光明中医. 2020;35(09):1275-9. | Non-clinic research (CM theory) |
| 彭修娟, 杨新杰, 许刚, 陈衍斌, 杨长花, 龚伟玲, et al. 基于整合药理学探讨清肺排毒汤治疗新型冠状病毒肺炎的功效及作用机制. 中国实验方剂学杂志. 2020;26(16):6-13. | Integrated pharmacology |
| 徐天馥, 贺成功, 杨坤. 基于网络药理学清肺排毒汤治疗新冠肺炎的物质基础及作用机制研究. 天然产物研究与开发. 2020;32(06):901-8. | Network pharmacology |
| 戴雅吉, 蒋磊, 高家荣, 郭明飞. 采用网络药理学和分子对接技术研究血必净注射液抗新型冠状病毒的作用机制. 中国医院药学杂志.1-8. | Network pharmacology and macromolecular docking |
| 文隆, 周志国, 姜迪譞, 黄康. 血必净注射液对重型新型冠状病毒肺炎患者炎症指标及病情转归的疗效观察. 中华危重病急救医学. 2020;32(04):426-9. | Non-comparison medication |
| 施烜, 魏娟, 刘美云, 金小红, 周焕平, 朱万莉, et al. 血必净注射液治疗新型冠状病毒肺炎的整体调控作用研究. 上海中医药杂志. 2020;54(04):46-52. | Network pharmacology |
| 曹如冰, 马清林, 徐倩娟, 段海婧, 杜丽东, 宁艳梅, et al. 新型冠状病毒肺炎诊治方案推荐医学观察期四种中成药对COVID-19的潜在共性机制分析. 中药药理与临床.1-16. | Network pharmacology |
| 李志军, 李银平, 王博超. 基于"三证三法"理论论治新型冠状病毒肺炎. 中华危重病急救医学. 2020(01):5-6-7. | Non-clinic research (CM theory) |
| 李思聪, 冯祥, 毕磊, 刘欣. 新型冠状病毒肺炎诊疗方案中成药选用分析与药理研究进展. 中药材.1-10. | Literature review |
| 李承羽, 张晓雨, 刘斯, 商洪才. 血必净注射液治疗新型冠状病毒感染的肺炎(COVID-19)证据基础及研究前瞻. 世界科学技术-中医药现代化. 2020;22(02):242-7. | Non-clinic research |
| 李春波, 苏韫, 刘永琦, 薛轩, 龚红霞, 李婷婷, et al. 清肺排毒汤治疗新型冠状病毒肺炎的中医理论及现代药理学机制. 中医杂志. 2020;61(15):1299-302. | Non-clinic research (CM theory) |
| 李红蓉, 常丽萍, 魏聪, 贾振华. 连花清瘟治疗新型冠状病毒肺炎的理论研究基础和临床疗效. 世界中医药. 2020;15(03):332-6. | Network pharmacology |
| 李长辉. 清肺排毒汤治疗新型冠状病毒肺炎浅析. 中国民间疗法. 2020;28(13):6-8. | Non-clinic research (CM theory) |
| 杜燕, 张选国. 清肺排毒汤治疗新型冠状病毒肺炎临床依据概述. 陕西中医. 2020;41(08):1016-9. | Non-clinic research (CM theory) |
| 杨猛, 杨少华, 杨眉, 游顶云. 中药连花清瘟治疗新型冠状病毒肺炎的系统评价. 中国药物评价. 2020;37(02):126-30. | Systematic review |
| 杨璞叶, 黄小正, 杨明博, 张鑫. 从病理学角度探讨清肺排毒汤联合克力芝治疗新冠肺炎的优势. 陕西中医药大学学报. 2020;43(03):1-4. | Pathology |
| 杨秀伟. 抗新型冠状病毒肺炎(COVID-19)的化湿败毒颗粒药味物质基础研究. 中国现代中药. 2020;22(05):672-89. | Non-clinic research (CM theory) |
| 杨默, 孙哲, 刘培杰. 清肺排毒方配合干扰素α治疗新型冠状病毒肺炎疗效分析. 深圳中西医结合杂志. 2020;30(08):29-30. | Combined Treatment (Qingfeipeidu plus interferon alpha) |
| 林嘉荣, 郑慰武, 曾贵兴, 林启展. 金花清感颗粒治疗新型冠状病毒肺炎网络药理学的研究. 中药材. 2020(08):2074-80. | Network pharmacology |
| 柳丽丽, 袁连方, 冯毅, 孙东, 刘文生, 王毅军, et al. 阿比多尔联合连花清瘟胶囊治疗新型冠状病毒肺炎的临床观察. 广东医学. 2020;41(12):1207-10. | Not targeted medication for this study |
| 段海婧, 龙晓宙, 杜丽东, 宁艳梅, 曹如冰, 任远. 清肺排毒汤对三种冠状病毒感染的SARS、MERS和COVID-19的潜在共性作用及机制探讨. 中药药理与临床.1-17. | Network pharmacology |
| 毛昀, 苏毅馨, 薛鹏, 李林潞, 朱世杰. 金花清感颗粒治疗新型冠状病毒肺炎作用机制探讨. 中药材.1-8. | Network pharmacology |
| 毛靖, 陆兔林. 从中医理论探讨清肺排毒汤对新型冠状病毒肺炎的治疗. 实用中医内科杂志.1-7. | Non-clinic research (CM theory) |
| 汪升早, 王华军, 陈鸿明, 岳玉, 卜凡进, 张雪梅. 连花清瘟胶囊和α-干扰素联合洛匹那韦/利托那韦治疗新型冠状病毒肺炎30例. 蚌埠医学院学报. 2020;45(02):154-5. | Not targeted medication for this study |
| 沈浮, 付中应, 吴泳蓉, 李玲, 赵昱东, 夏雨, et al. 基于网络药理学及高通量分子对接研究金花清感颗粒中结合SARS-CoV-2特定靶蛋白的活性化合物干预COVID-19的潜在分子机制. 世界科学技术-中医药现代化. 2020;22(03):622-31. | Network pharmacology and macromolecular docking |
| 沈爱明, 张伟, 吴卓, 王文龙, 花佳佳. 清肺排毒汤治疗新型冠状病毒肺炎的中医理论分析. 辽宁中医杂志. 2020;47(03):106-8. | Non-clinic research (CM theory) |
| 漆国栋, 漆伟, 江琼, 申开琴, 张雪, 张练. 连花清瘟结合西医方案对新冠肺炎普通型患者疗效的系统评价. 中医药临床杂志. 2020;32(07):1195-9. | Systematic review |
| 王一竹, 柳芳, 张相林. 新型冠状病毒肺炎诊疗方案中的口服中成药应用综述. 中国医院用药评价与分析. 2020;20(03):257-61+67. | Literature review |
| 王君平. 抗疫，中医药添加新力量. 农村·农业·农民(A版). 2020(04):51-2. | Non-clinic research |
| 王恩成, 唐琳, 徐奎, 冯全生. 清肺排毒汤治疗75例新型冠状病毒肺炎轻型与普通型酶学指标改善的疗效评价. 中药与临床. 2020;11(01):3-5. | Non-comparison medication |
| 王朝, 尹梦碟, 方泓, 张天嵩, 袁敏. 基于中医名家经典浅析新冠肺炎中清肺排毒汤的运用. 中医药文化. 2020;15(04):11-6. | Non-clinic research (CM theory) |
| 王林, 杨志华, 张浩然, 于航星, 杨康, 付宝慧, et al. 连花清瘟治疗新型冠状病毒(2019-nCoV)肺炎网络药理学研究与初证. 中药材. 2020(03):772-8. | Network pharmacology |
| 王毅, 李翔, 张俊华, 薛睿, 钱竞扬, 张晓慧, et al. 基于网络药理学的宣肺败毒汤治疗新型冠状病毒肺炎机制研究. 中国中药杂志. 2020;45(10):2249-56. | Network pharmacology |
| 王汉, 宋红新, 王敦方, 马旭冉, 邹迪新, 苗金雪, et al. 基于网络药理学和分子对接探讨宣肺败毒方治疗COVID-19抗病毒作用的分子机制. 海南医学院学报.1-13. | Network pharmacology and macromolecular docking |
| 王汉, 王敦方, 宋红新, 马旭冉, 邹迪新, 苗金雪, et al. 从《伤寒论》六经辨证探讨抗新冠肺炎“清肺排毒汤”的组方规律. 海南医学院学报.1-8. | Non-clinic research (CM theory) |
| 王法财, 沈炳香, 何春远, 赵为陈, 聂松柳. 连花清瘟颗粒对新型冠状病毒肺炎的临床疗效及其机制的网络药理学研究. 中药药理与临床. 2020;36(02):93-101. | Non-comparison patients group |
| 王淑霞, 李明阳, 陈雪莲, 马明艳, 胡继宏. 连花清瘟联合西医治疗新型冠状病毒肺炎临床疗效的Meta分析. 中草药. 2020;51(14):3763-9. | Meta analysis |
| 王饶琼, 杨思进, 谢春光, 沈其霖, 李敏清, 雷枭, et al. 清肺排毒汤治疗新型冠状病毒肺炎的临床疗效观察. 中药药理与临床. 2020;36(01):13-8. | No-comparison patients group |
| 田晓玲, 华川, 张艳, 赵勇. 连花清瘟胶囊防治新型冠状病毒肺炎研究进展. 辽宁中医药大学学报. 2020;22(08):184-7. | Non-clinic research |
| 田朝晖, 向建军, 葛舰, 秦开烈, 李玉英, 王奎, et al. 清肺排毒汤治疗新型冠状病毒肺炎理论分析与临床实践. 世界中医药. 2020;15(04):497-501. | Network pharmacology |
| 田毅萍, 高玉林. 浅析清肺排毒汤治疗新冠肺炎的理论依据及作用机制. 中国医药导刊. 2020;22(05):289-93. | Non-clinic research (CM theory) |
| 秦凤凤, 李时超, 孙亚宁, 刘英, 彭顺林. 基于网络药理学与分子对接探索血必净治疗新型冠状病毒感染所致的ARDS的分子机制. 中药药理与临床. 2020;36(03):21-8. | Network pharmacology and macromolecular docking |
| 秦子楠, 王梦昕, 史楠楠, 王燕平, 翟华强. 基于文献分析的细辛在清肺排毒汤中合理应用. 中国中药杂志. 2020;45(07):1515-20. | Non-clinic research (CM theory) |
| 程德忠, 李毅. 连花清瘟颗粒治疗54例新型冠状病毒肺炎患者临床分析及典型病例报道. 世界中医药. 2020;15(02):150-4. | Non-comparison patients group |
| 程玉峰, 夏友宏, 周大勇, 杨从鑫. 亳州市84例新型冠状病毒肺炎的辨证论治分析. 安徽中医药大学学报. 2020;39(02):9-11. | Non-standard research design |
| 耿立梅, 孙庆臣, 郭登洲, 梅建强, 耿少怡, 宿英豪, et al. 对清肺排毒汤《河北省中医药救治新冠肺炎专家共识八条》之解读. 河北中医药学报. 2020;35(03):51-4. | Non-clinic research (CM theory) |
| 芮剑. 结合五运六气对新型冠状病毒肺炎及清肺排毒汤的思考. 甘肃医药. 2020;39(03):257-8. | Non-clinic research (CM theory) |
| 范建新, 秦雪梅, 李震宇. 基于网络药理学和分子对接技术的款冬花在清肺排毒汤治疗新型冠状病毒肺炎（COVID-19）中的作用分析. 中草药. 2020;51(09):2317-25. | Network pharmacology and macromolecular docking |
| 范毓慧, 刘晓节, 秦雪梅, 杜冠华. 防治COVID-19的含黄芩方药分析及其网络药理作用机制研究进展. 中药药理与临床.1-19. | Network pharmacology |
| 范逸品, 王燕平, 马艳, 赵晨, 张华敏. 从新型冠状病毒肺炎的寒疫病机探析清肺排毒汤的组方机制. 中国实验方剂学杂志. 2020;26(16):1-5. | Non-clinic research |
| 董丽, 杨雪, 张林松, 李亚琴, 王饶琼, 杨思进. 清肺排毒汤中西医结合辨证论治新型冠状病毒肺炎2例. 中药药理与临床. 2020;36(02):55-8. | Case study |
| 蒋谦谦, 陈新宇. 基于方证辨证对清肺排毒汤快速有效治疗新型冠状病毒肺炎的思考. 中医杂志. 2020;61(14):1204-6. | Non-clinic research |
| 薛伯寿, 姚魁武, 薛燕星. “清肺排毒汤”快速有效治疗新型冠状病毒肺炎的中医理论分析. 中医杂志. 2020;61(06):461-2. | Non-clinic research (CM theory) |
| 许冬玉, 许玉龙, 王至婉, 吕雅丽, 朱红磊, 宋婷. 基于网络药理学研究清肺排毒汤治疗新型冠状病毒肺炎的作用机制. 中药药理与临床. 2020;36(01):26-32. | Network pharmacology |
| 许禄华, 李彦荣, 郑丹如, 邵宗钫, 闻思齐, 林丰夏, et al. 基于“因时制宜”探讨新型冠状病毒肺炎不同阶段的处方用药规律. 中国实验方剂学杂志.1-11. | Non-clinic research (CM theory) |
| 谢铱子, 钟彩婷, 纪树亮, 黄宝仪, 黄慧婷, 詹少锋, et al. 基于网络药理学及分子对接技术探讨化湿败毒方治疗新型冠状病毒肺炎的分子机制. 中药药理与临床. 2020;36(03):28-35. | Network pharmacology and macromolecular docking |
| 谢鸣. 对中医药防治新型冠状病毒肺炎清肺排毒汤制方及运用的思考. 中医杂志. 2020;61(13):1105-9. | Non-clinic research (CM theory) |
| 贾振华. 网络病理论指导新型冠状病毒肺炎证治探讨. 中国实验方剂学杂志. 2020;26(12):18-22. | Network pharmacology |
| 贾振华, 李红蓉, 常丽萍, 魏聪. 中医学应对疫病的历史回顾与思考. 中国实验方剂学杂志. 2020;26(11):1-7. | Non-clinic research |
| 赖庆来, 梁爱武, 何妙仪, 黄小玉, 吴伟泳. 化湿败毒方治疗新型冠状病毒肺炎的药理学机制探讨和网络药理学研究. 天然产物研究与开发. 2020;32(06):909-19. | Network pharmacology |
| 赖慧, 殷文贤, 赵福兰, 陈立, 杨思进, 米晓琴. 自研“新冠0号“”新冠1号”防治新型冠状病毒肺炎效果与分析. 中国药业. 2020;29(07):9-11. | Non-standard research design |
| 赵静, 田赛赛, 杨健, 刘剑锋, 张卫东. 清肺排毒汤治疗新型冠状病毒肺炎机制的网络药理学探讨. 中草药. 2020;51(04):829-35. | Network pharmacology |
| 路志正, 路喜善. 清肺排毒汤彰显中医药抗疫疗效与自信. 中医杂志. 2020;61(10):833-4. | Non-clinic research |
| 邱保国. 中医治疗新型冠状病毒肺炎诠释. 中医研究. 2020;33(09):1-6. | Non-clinic research |
| 郑雅, 刘志强, 朱晓芹, 王博龙. 基于网络药理学和分子对接探讨血必净注射液治疗新型冠状病毒肺炎(COVID-19)的作用机制. 中国比较医学杂志. 2020;30(07):57-64. | Network pharmacology and macromolecular docking |
| 郭程程, 焦华琛, 李运伦. 中医“扶正祛邪”治则在“三药三方”治疗新冠肺炎中的体现. 辽宁中医药大学学报.1-11. | Non-clinic research (CM theory) |
| 鄢海燕, 邹妍, 邹纯才. 基于网络药理学和分子对接技术分析清肺排毒汤治疗COVID-19的机制. 南方医科大学学报. 2020;40(05):616-23. | Network pharmacology and macromolecular docking |
| 金世元. 自古防疫方药众,清肺排毒汤和而不同. 中医杂志. 2020;61(10):835-6. | Non-clinic research (CM theory) |
| 靳晓杰, 关瑞宁, 毛建军, 王燕如, 王菲, 李潮新, et al. 基于计算机辅助药物设计的清肺排毒汤多靶点系统治疗新型冠状病毒肺炎(COVID-19)物质基础探究. 中草药. 2020;51(08):1984-95. | Computer based pharmacology |
| 韩茂之, 李莎莎, 李静, 李宪超, 高林林, 鹿艳, et al. 清肺排毒汤致新型冠状病毒肺炎患者高钾血症. 药物不良反应杂志. 2020;22(06):375-6. | Case study |
| 高燕菁. 《新型冠状病毒肺炎诊疗方案》中医临床治疗首选方剂——清肺排毒方. 家庭中医药. 2020;27(05):37-9. | Non-clinic research |
| 高燕菁. 治疗重型和危重型新冠肺炎推荐中药注射剂——血必净. 家庭中医药. 2020;27(05):40-1. | Non-clinic research |
| 龚普阳, 郭瑜婕, 李晓朋, 王楠, 顾健. 基于网络药理学与分子对接技术的金花清感颗粒防治新型冠状病毒肺炎的潜在药效物质研究. 中草药. 2020;51(07):1685-93. | Network pharmacology and macromolecular docking |
| 中医药有效方剂筛选研究取得阶段性进展——试点省份临床观察显示：清肺排毒汤治疗总有效率可达90%以上. 中医药通报. 2020;19(01):9. | Non-clinic research |
| 治疗新冠肺炎，中药为啥有作用. 晚晴. 2020(02):88. | Non-clinic research |

**Supplementary Material 3: Re-calculation of the statistical significance of disease severity progression reported in Cheng 2020**

Translation of Cheng 2020’s paper is as follow:

‘计数资料采用卡方检验或Fisher 精确概率法’

‘Count data will be tested by χ2 or Fisher Exact. ‘

‘两组患者治疗过程中转重型情况分析 治疗组 51 例,治疗过程中转重型 4 例(7.8%);对照组

51 例,转重型 11 例(21.6%),组间比较差异有统计学意义(P<0.05)。’

‘Progression to severe type during the treatment: treatment group, 51 patients, 4 (7.8%) progressed; comparator group 51 patients, 11 (21.6%) progressed. The inter-group difference has statistical meaning (P<0.05).’

Below we put the numbers in to a 2x2 contingency table and performed the χ2 test by hand:

|  | Progressed | Not progressed | Total |
| --- | --- | --- | --- |
| Intervention group | 4 | 47 | 51 |
| Comparator group | 11 | 40 | 51 |
| Total | 15 | 87 | 102 |

$$\chi2=\frac{{(4\times40-47\times11)}^{2}\times102}{51\times51\times15\times87}=3.830$$

From χ2 distribution table, for degree of freedom = 1, we can see that 3.830 is between 2.706 (when p=0.1) and 3.841 (when p=0.05), so the p value should between 0.05 and 0.1. It is 0.0503 to be exact.

Similarly, we performed the Fisher Exact test, and the result is p= 0.0911.

**Supplementary Material** 4**: Data inconsistency in Yu 2020**

Translation of Yu 2020’s paper is as follow:

‘经7d 治疗，观察组转重症18 例（其中8 例转火神山医院），死亡1 例；对照组转重症38 例（其中12 例转火神山医院），死亡2 例；转重症及死亡均归为无效统计。两组治疗有效率及转重症率对比，均具有统计学差异（P<0.05）。见表2’

‘After 7 days of treatment, observational group had 18 patients turned severe (among them, 8 were transferred to Huoshenshan Hospital), 1 death. Comparator group had 38 patients turned severe (among them, 12 were transferred to Huoshenshan Hospital), 2 deaths. Progression to severe type and death are all counted toward “inefficacy”. There are statistically significant differences between the groups in the rate of efficacy and progression to severe type (p<0.05). See Table 2’

Part of table 2 is shown below, which does not match the numbers above.

| Group | Inefficacy | Progression to severe type |
| --- | --- | --- |
| Observation group (n=147) | 28 | 21 |
| Comparator group (n=148) | 52 | 35 |

We wrote to the corresponding author on May 2 but received no response.

**Supplementary Material 5: TCM symptom scoring in studies**

| Code of study | Scoring standard | Score of this study |
| --- | --- | --- |
| Cheng, 2020 | 1. Primary outcomes (fever, fatigue, cough): resolved 0 score, unresolved 1 score 2. (pre-treatment - post-treatment)/pre-treatment x 100% = scoring reduce rate 3. Valid treatment: scoring reduce rate > 30%; invalid treatment: scoring reduce rate≤30% 4. Valid treatment/ total number of patients x 100% = Total valid rate of intervention | Total valid rate of intervention:  TG: 44/51 (86.3%)  CG: 35/51 (68.6%)  P=0.033 |
| Duan, 2020 | According *to The Guidance of New CHM Clinical research (pilot edition)*^1^   1. Symptom resolved: 0 score 2. Mild symptom: 1 score 3. Moderate symptom: 2 score 4. Severe symptom: 3 score 5. Total score: 0 to 69 score 6. There is no difference of weight of each symptom, the final score of intervention is the summation of scores of all symptoms | TG:  Pre-treatment: 15.57±4.32  Post-treatment: 10.02±3.80  (P<0.01)  CG:  Pre-treatment: 16.15±4.42  Post-treatment: 11.39±2.73  (P<0.01)  Total P<0.05 |
| Yu, 2020 | According *to The Guidance of New CHM Clinical research (pilot edition)*^1^   1. To assess symptoms like fever, fatigue, cough, dry and sore throat, blocked chest, etc. 2. Symptom resolved: 1 score 3. Mild symptom: 2 score 4. Moderate symptom: 3 score 5. Severe symptom: 4 score 6. To assess one time before and after treatment  1. Total score reduced ≥70%, significantly valid treatment, symptoms almost relieved 2. Total score reduced ≥ 30%, valid treatment, symptoms relieved at some extent 3. Total score reduced < 30%, invalid treatment, symptoms unrelieved | TG:  Pre-treatment: fever (2.37±0.43), fatigue (2.52±0.41), cough (3.45±0.47), dry and sore throat (3.59±0.44), blocked chest (2.47±0.52)  Post-treatment: fever (0.56±0.14), fatigue (0.78±0.25), cough (1.39±0.42), dry and sore throat (0.78±0.25), blocked chest (1.14±0.72)  (P<0.05)  CG:  Pre-treatment: fever (2.45±0.49), fatigue (2.58±0.44), cough (3.42±0.58), dry and sore throat (3.53±0.49), blocked chest (2.53±0.45)  Post-treatment: fever (1.12±0.32), fatigue (1.12±0.28), cough (2.56±0.51), dry and sore throat (2.12±0.56), blocked chest (1.57±0.53)  (P<0.05)  Total P< 0.05 |

Reference:

1. Zheng X. Guiding Principles of Clinical Research on New Drugs of Traditional Chinese Medicine. Beijing: Science and Technology Press; 2002
